# Bioinformatic analysis of membrane and associated proteins in murine cardiomyocytes and human myocardium

**DOI:** 10.1038/s41597-020-00762-1

**Published:** 2020-12-01

**Authors:** Shin-Haw Lee, Sina Hadipour-Lakmehsari, Da Hye Kim, Michelle Di Paola, Uros Kuzmanov, Saumya Shah, Joseph Jong-Hwan Lee, Thomas Kislinger, Parveen Sharma, Gavin Y. Oudit, Anthony O. Gramolini

**Affiliations:** 1 Translational Biology and Engineering Program, Ted Rogers Centre for Heart Research, Toronto, Ontario M5G 1M1 Canada; 2 Department of Physiology, Faculty of Medicine, University of Toronto, Toronto, Ontario M5S 1M8 Canada; 3 Department of Medicine, University of Alberta, Edmonton, Alberta T6G 2R3 Canada; 4 Mazankowski Alberta Heart Institute, Edmonton, Alberta T6G 2B7 Canada; 5 Princess Margaret Cancer Research Centre, Toronto, Ontario M5G 1L8 Canada; 6 Department of Medical Biophysics, Faculty of Medicine, University of Toronto, Toronto, Ontario M5G 1L7 Canada; 7 Present Address: Department of Cardiovascular & Metabolic Medicine, University of Liverpool, Liverpool, L69 3GE UK

**Keywords:** Proteomics, Mechanisms of disease

## Abstract

In the current study we examined several proteomic- and RNA-Seq-based datasets of cardiac-enriched, cell-surface and membrane-associated proteins in human fetal and mouse neonatal ventricular cardiomyocytes. By integrating available microarray and tissue expression profiles with MGI phenotypic analysis, we identified 173 membrane-associated proteins that are cardiac-enriched, conserved amongst eukaryotic species, and have not yet been linked to a ‘cardiac’ Phenotype-Ontology. To highlight the utility of this dataset, we selected several proteins to investigate more carefully, including FAM162A, MCT1, and COX20, to show cardiac enrichment, subcellular distribution and expression patterns in disease. We performed three-dimensional confocal imaging analysis to validate subcellular localization and expression in adult mouse ventricular cardiomyocytes. FAM162A, MCT1, and COX20 were expressed differentially at the transcriptomic and proteomic levels in multiple models of mouse and human heart diseases and may represent potential diagnostic and therapeutic targets for human dilated and ischemic cardiomyopathies. Altogether, we believe this comprehensive cardiomyocyte membrane proteome dataset will prove instrumental to future investigations aimed at characterizing heart disease markers and/or therapeutic targets for heart failure.

## Introduction

Cell surface or membrane proteins serve as excellent candidates and potential diagnostic and therapeutic targets given their importance in regulating spontaneous contractions and excitation-contraction coupling of cardiomyocytes^1,2^. Additionally, membrane proteins in the mammalian heart act as important signaling receptors, metabolite transporters, enzymes, cell-cell adhesion anchors, and electrical propagation regulators^3^. Therefore, a comprehensive understanding of all cardiac membrane proteins is essential to elucidate and characterize the physiology and pathophysiology of human heart diseases. However, in-depth analysis and identification of the membrane proteome of cardiomyocytes remains poorly characterized due to their hydrophobic nature and relatively low abundance^4^.

Recent advancements in proteomic technologies have allowed significant improvements in membrane protein proteome discovery and topology resolution^5,6^. Specifically, advancements in mass spectrometry-based shotgun proteomics have allowed successful identification of less abundant membrane proteins, resulting in substantial improvements in representation of membrane proteins in proteomic datasets^6–8^. Therefore, identification of a more complete membrane proteome in the heart has become possible and will contribute to a more detailed understanding of signaling networks, pathways, and protein-protein interactions in cellular biology of the heart.

At the cellular level, the molecular basis and mechanisms of impaired ventricular function in heart failure patients remain poorly understood. Alterations in membrane protein expression levels have been linked largely to depressed Ca^2+^ signaling and impaired relaxation in heart failure^9^, suggesting important functional relevance of membrane proteins in cardiac muscle biology, thus representing excellent therapeutic targets that modulate cellular signaling and function in failing cardiac muscle. To date, the majority of cardiac proteomic and biochemical studies have focused on abundant soluble proteins. In this study, we carefully investigated membrane proteins identified previously^10^, incorporating publicly available microarray dataset, tissue expression profiles, and informatics that resulted in the identification of 173 previously uncharacterized, intrinsic cardiac-enriched membrane-associated proteins from human fetal and mouse neonatal ventricular cardiomyocytes. As evidence of the utility of this set, we applied a non-biased, systematic ranking strategy to all candidates and selected several highest ranked proteins to validate including FAM162A, MCT1, and COX20 as potentially novel regulators of cardiac function.

## Results

### Cardiac proteomic data analysis uncovers poorly characterized, cardiac-enriched membrane proteins

**Fig. 1:**
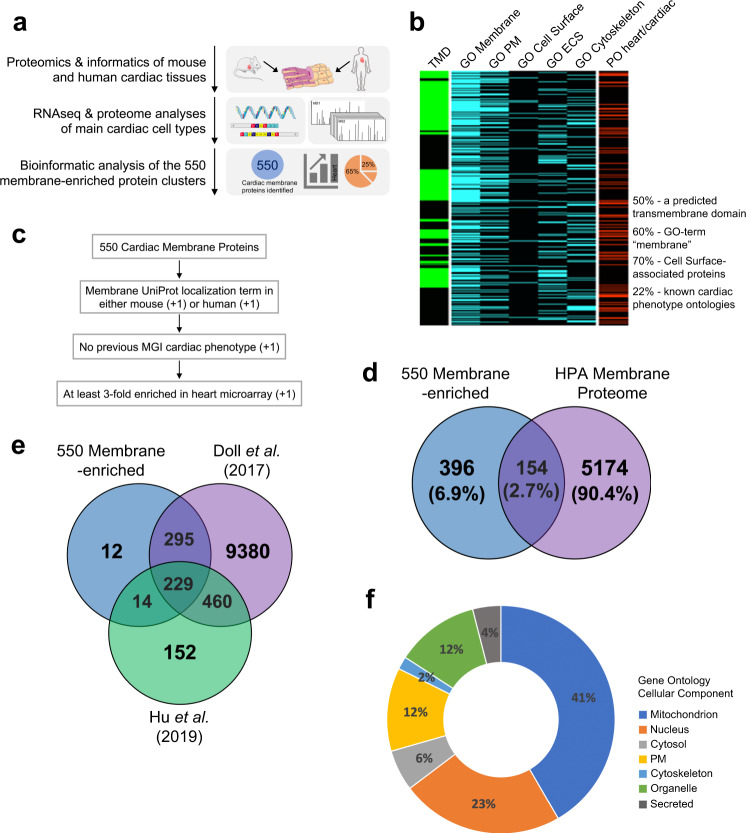
Membrane proteomic analysis of human fetal and murine ventricular cardiomyocytes. (**a**) Schematic diagram of experimental workflow that resulted in the identification of 550 membrane-enriched protein clusters in human and mouse cardiomyocytes. (**b**) Heatmap depicting Gene Ontology (GO) term associated distribution and cardiac/heart phenotype ontologies of the 550 membrane-enriched protein clusters. (**c**) Schematic diagram of the ranking strategy applied to the 550 protein clusters to identify previously uncharacterized cardiac-enriched membrane proteins. (**d**) Venn diagram depicting overlap of protein coverage between our membrane-enriched proteome from human and mouse ventricular cardiomyocytes and the Human Protein Atlas Membrane Proteome. (**e**) Venn diagrams depicting overlap of protein coverage between our membrane-enriched proteome from human and mouse ventricular cardiomyocytes and the monkey (*Rhesus macaque*) heart proteome from Hu *et al*.^13^ and human heart proteome from Doll *et al*.^14^. (**f**) Distribution of the 550 membrane-enriched protein clusters across various subcellular organelles based on their GO cellular component classification. Detailed ranking criteria and step-by-step ranking of all candidates were uploaded to figshare (10.6084/m9.figshare.11844972.v12).

To identify the proteome of cell-surface/membrane associated and soluble proteins of human and mouse ventricular cardiomyocytes, we optimized previously the membrane enrichment of human fetal and mouse neonatal ventricular cardiomyocytes to enrich for cell surface, membrane, and membrane-associated proteins via cationic colloidal silica beads^10,11^. Quantitative MudPIT analysis had identified 2,762 human and 3,033 mouse proteins with stringent filtering (<1% FDR) of peptide and protein levels. A subsequent comparative QSpec proteomic analysis revealed 555 orthologue proteins were significantly enriched in the membrane fractions compared to the membrane-depleted counterpart. A recent update of gene names realigned the data to 550 proteins since 5 protein IDs were amalgamated with existing protein accessions (Fig. 1a). Our earlier studies focused on two of these proteins, TMEM65 and REEP5, in detailed biochemical analyses, identifying their specific roles in regulating cardiac conduction and cardiac muscle sarcoplasmic reticulum organization and function, respectively^10,12^. Here, we performed a detailed analysis of all 550 identified cardiac membrane proteins and showed that GO term-associated classifications demonstrated 50% of the identified protein clusters had a predicted transmembrane domain and 60% of them contained the GO term “membrane” (Fig. 1b). Moreover, 70% of the 550 protein clusters were classified as cell surface-associated proteins and only 20% of them were linked to known cardiac phenotype ontologies based on available forward genetics studies in mice in the MGI database (Fig. 1b). All 550 proteins were each scored and ranked on a set of non-biased, defined criteria (Fig. 1c and full ranking criteria listed in Supplemental Table 1) to prioritize proteins that had no previous association to cardiac function and were enriched >3-fold within cardiac tissue compared to other tissues, according to publicly available microarrays (Supplemental Table 1; individual worksheets represent rank ordered analysis of the 550 membrane associated protein clusters in various subcellular domains). Based on this analysis, this strategy resulted in 173 of these membrane-associated proteins that would be classified as previously uncharacterized, cardiac-enriched membrane-associated proteins. Hierarchical clustering of the 550 membrane-associated protein clusters demonstrated distinct clusters of cardiac enrichment across various healthy human tissues (Supplemental Fig. 1a).

We next mapped these 550 membrane enrichment proteins to the Human Protein Atlas ‘Membrane Proteome’; where approximately 28% of all human protein coding genes are predicted to harbor at least one transmembrane domain (Fig. 1d). This analysis showed that 154 of our identified cardiac-enriched membrane proteins were present in the Membrane Proteome, leaving 396 out of the 550 protein clusters potentially novel membrane protein products. Similarly, we compared our list of 550 proteins against recent studies of whole tissue lysates from monkey heart proteome by Hu *et al*.^13^ and the human heart proteome by Doll *et al*.^14^ (Fig. 1e). Of note, 229 cardiac-enriched membrane proteins were also identified in both of these studies with 12 unique proteins identified only in our analysis (Fig. 1e).

Next, we assessed the organelle distribution of all “membrane-associated” candidates identified by our proteomics and informatics analyses. We analyzed their subcellular localizations based on their Gene Ontology cellular component (GOCC) classification (Fig. 1f) and observed that 228/550 (41%) of all candidate proteins were identified as *mitochondrial* in origin which was expected as cardiomyocytes are highly oxidative and metabolically active. Interestingly, 125/550 (23%) of identified membrane-associated proteins were classified as localized to the *nucleus*, likely suggesting the dynamic roles of nuclear membrane proteins in regulating gene expression, cell mobility, and DNA damage repair in cardiomyocytes^15,16^ (Fig. 1f). Moreover, 63/550 (12%) of the 550 membrane associated protein clusters identified were classified as ‘*cell surface’* or ‘*plasma membrane’* in origin and another 65/550 (12%) were attributed to other organelles including the *endoplasmic reticulum*, *golgi apparatus*, *peroxisomes*, and *lysosomes*. Interestingly, of much less abundance, 6% (32/550) and 4% (22/550) of protein clusters were classified as *cytosolic* or proteins in the *secretory pathway*, respectively, suggesting the identification of membrane-associated cytosolic and secreted proteins in our proteomic analysis. However, it is important to acknowledge that Gene Ontology classification has limitations, further detailed biomolecular studies obviously would be needed to determine precise subcellular localization and function in the cell.

### Identification of organelle-specific, previously uncharacterized cardiac membrane proteins

**Fig. 2:**
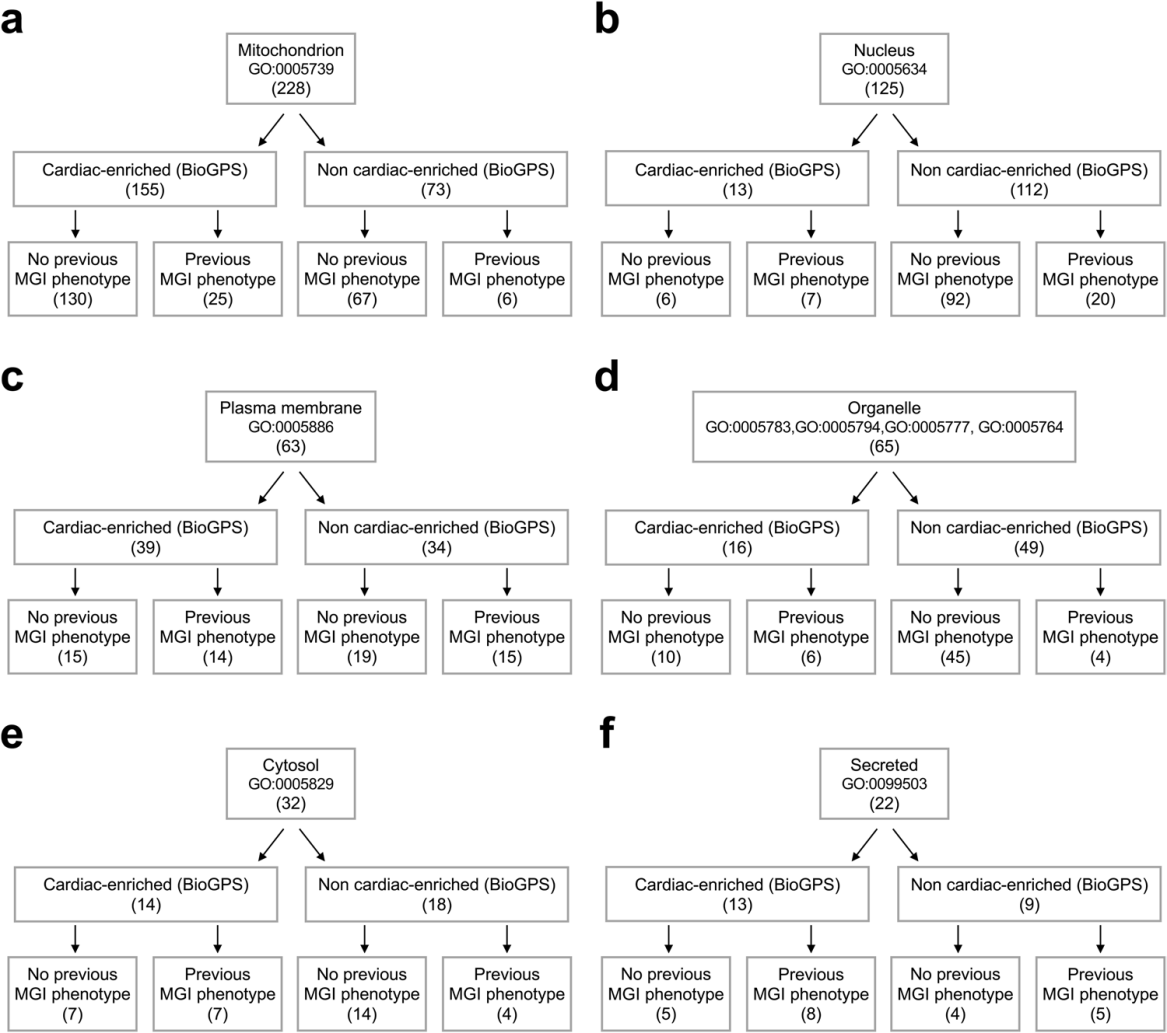
Analysis of cardiac membrane proteins within various subcellular domains. Consort diagrams showing classification of the 550 membrane-enriched protein clusters and analyses of cardiac enrichment and phenotypic novelty based on publicly available BioGPS microarray data and MGI phenotypic analysis, respectively in (**a**) mitochondrion, (**b**) nucleus, (**c**) plasma membrane, (**d**) other organelles (ER, golgi apparatus, peroxisomes, lysosomes), (**e**) cytosol, and (**f**) the secretory pathway.

Next, we aimed to identify highly scored cardiac-enriched protein clusters that have not been investigated to date in the heart across various organelles in the myocyte. Based on previous GOCC classification, we had identified 228 mitochondrial membrane proteins, 125 nuclear membrane proteins, 63 plasma membrane associated proteins, 65 ER, golgi apparatus, peroxisome, lysosome-associated membrane proteins, 32 cytosolic, and 22 secreted membrane associated proteins (Fig. 1f). Our bioinformatic analysis (Fig. 2) showed that 155 (68%) mitochondrial membrane-associated proteins were at least 3-fold enriched within cardiac tissue according to publicly available BioGPS microarrays; 130 of which were further classified as ‘novel’ cardiac membrane proteins with no previously reported MGI cardiac related phenotype. Similarly, 10% of all identified nuclear membrane associated proteins were found cardiac-enriched by BioGPS, and 6 of the 13 cardiac-enriched nuclear membrane associated proteins were reported with no previous MGI heart related phenotype (Fig. 2b).

Detailed analysis of the plasma membrane-associated protein clusters in Fig. 2c showed that 39 (62%) of which were found 3-fold enriched within cardiac tissue, 15 of these were further classified as novel PM related cardiac-enriched membrane proteins with no phenotype ontology. Similar informatic analysis of ER, golgi apparatus, peroxisome, and lysosome-related membrane proteins showed that 16 (25%) organellar proteins were considered cardiac-enriched, 10 of which were further classified as novel cardiac-enriched organellar membrane-associated proteins with no previous reported cardiac phenotype (Fig. 2d). Interestingly, AFR3 was solely identified in our membrane proteomic analysis of human and mouse cardiomyocytes. Additionally, 14 (44%) cytosolic and 13 (59%) secreted protein clusters were found to be cardiac-enriched (Fig. 2e,f). Specifically, 7 cytosolic membrane-associated proteins had no previously reported MGI heart related phenotype, and 5 secreted membrane-associated proteins were found with no reported MGI heart phenotype.

**Fig. 3:**
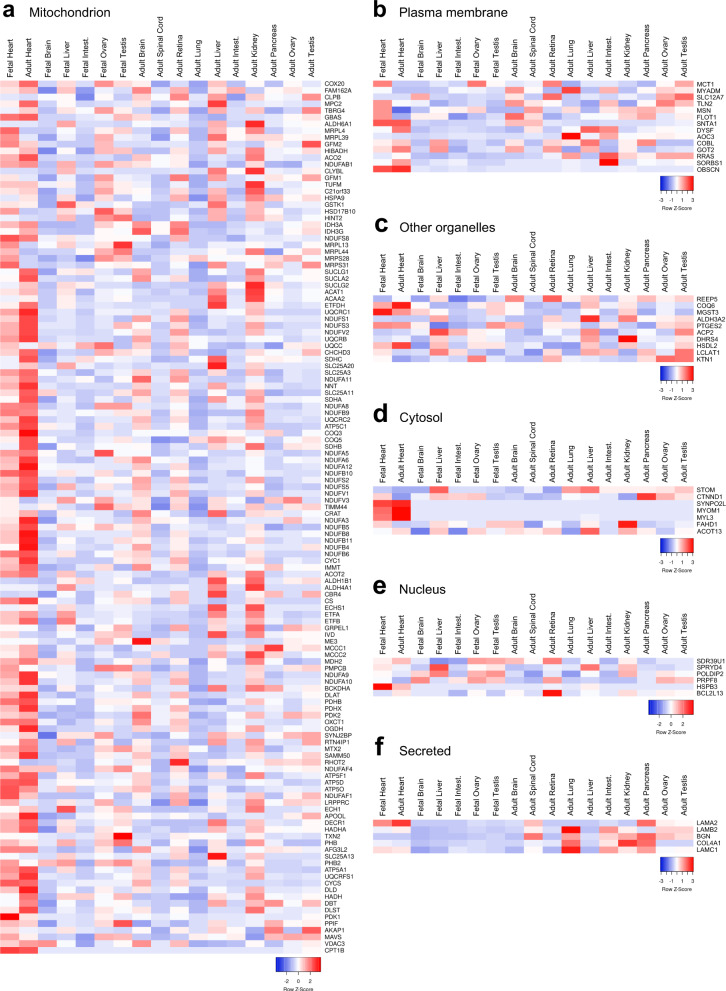
Transcriptomic analysis of novel cardiomyocyte-enriched membrane proteins shows cardiac enrichment across various tissues. Heatmaps showing mRNA transcript levels of 173 novel cardiac-enriched membrane proteins across clinically defined healthy human tissues; mRNA transcript data were obtained from Human Proteome Map and are presented according to their subcellular classifications in (**a**) mitochondrion, (**b**) plasma membrane, (**c**) other organelles (ER, golgi apparatus, peroxisomes, lysosomes), (**d**) cytosol, (**e**) nucleus, and (**f**) the secretory pathway. All source data input and normalized output files were uploaded to figshare (10.6084/m9.figshare.11844972.v12).

Next, tissue-wide transcriptomic analysis of these previously uncharacterized, cardiac-enriched membrane proteins was performed across non-diseased human tissues. Specifically, we employed mRNA transcript data from the Human Proteome Map^17^ and showed dominant cardiac expression across various organ tissues for each organellar classification (Fig. 3). Specifically, the gene level expression matrix was downloaded and normalized to total expression across all tissues^17^. Figure 3a shows predominant mRNA expression in the fetal and adult heart of most of the 130 mitochondrial related membrane proteins. Notably, COQ3, NDUFB8, NDUFB11, PDHB, ATP5D, APOOL, PDK1, and CPT1B showed nearly exclusive expression in the heart. Moreover, dominant cardiac expression of MRL4, MRPS31, and PDK1 in the fetal heart compared to adult heart was observed. Conversely, expression of SUCLG1, SUCLG2, SDHA, COQ3, NDUFA3, and NDUFB4 was seen in the adult heart compared to fetal heart (Fig. 3a). Heat maps of the plasma membrane proteins showed proteins such as MCT1, DYSF and SORBS1 were enriched in the heart with some expression across the other tissues while OBSCN was detected only in the heart (Fig. 3b). Analysis of the 10 organellar (ER, golgi, peroxisomes, lysosomes) (Fig. 3c), 7 cytosolic (Fig. 3d), 6 nuclear (Fig. 3e), and 5 secreted (Fig. 3f) membrane-associated proteins showed transcript expression across various healthy human tissues. Notably, SYNPO2L, MYOM1, MYL3 showed restricted expression to only the cytosol in the heart (Fig. 3d). However, it is important to acknowledge that transcriptomic data must be interpreted carefully due to the poor correlation between mRNA expression level and the abundance of its associated protein product. To circumvent this issue, we applied a defined set of non-biased criteria to prioritize the candidate proteins for follow-up experiments (Supplemental Table 1). This strategy equally evaluated each criterion in our rank-ordered analysis of all candidates and resulted in a total of 18 highest ranked membrane proteins where existing data demonstrated their subcellular localization to both human and mouse cardiomyocyte membranes in the cell with no previous connection to cardiac function, and are least 3-fold enriched in the heart (Supplemental Table 1). Interestingly, many of the abovementioned cardiac preferential proteins were not among the 18 highest ranked candidate proteins due to non-membrane subcellular localization and/or previous association with cardiac function. These cardiac enriched proteins are quite likely of great interest in future studies to further our understanding of their precise role in heart, although they did not reach a high enough criterion weighting in our particular evaluation.

Nevertheless, transcriptomic analysis of the 67 cardiac-enriched membrane associated proteins with previous MGI cardiac phenotype further demonstrated the utility and potential of our dataset (Supplemental Fig. 2a–f). Specifically, mutations in cell surface protein DSC2 have been linked to arrhythmogenic and hypertrophic cardiomyopathies^18–21^ while POPDC2 has been shown to be essential for heart muscle development^22,23^. Deletion of cardiac cytosolic protein CTNNA3 in the mouse has been associated with dilated cardiomyopathy and actomyosin dysregulation in the heart^24,25^. Lastly, detailed transcriptomic breakdown of the non-cardiac-enriched protein distributions were also analyzed and shown in Supplemental Figs. 3, 4a–f for comparison.

### FAM162A, MCT1, and COX20 are evolutionarily conserved, cardiac ventricular-enriched membrane proteins

**Fig. 4:**
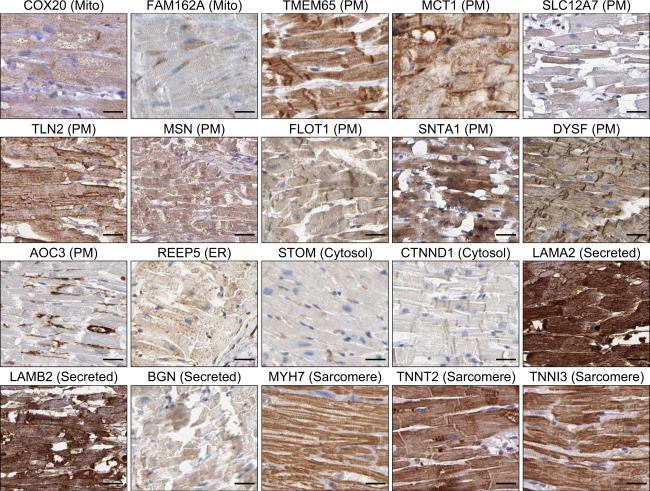
Immunohistochemistry analysis of top ranked cardiac-enriched candidates. Immunohistochemistry analysis of the top ranked candidates in each subcellular classification (Mitochondrion, plasma membrane, other organelles (ER, golgi apparatus, peroxisomes, lysosomes), cytosol, and the secretory pathway in healthy human ventricular tissues from the Human Protein Atlas. Immunohistochemical stain intensity was assessed independently as either *weak*, *moderate* or *strong* and reported as protein expression score (PES). COX20, moderate PES; FAM162A, weak PES; Tmem65, moderate PES; MCT1, moderate PES; SLC12A7, moderate PES; TLN2, moderate PES; MSN, moderate PES; FLOT1, moderate PES; SNTA1, moderate PES; DYSF, moderate PES; AOC3, negative PES; REEP5, moderate PES; STOM, weak PES; CTNND1, moderate PES; LAMA2, strong PES; LAMB2, strong PES; BGN, moderate PES; MYH7, strong PES; TNNT2, strong PES; TNNI3, strong PES. Scale bar, 25μm. All images shown are representative of approximately 5 distinct regions of interest (ROIs) assessed per sample, n = 3 independent patient tissue blocks.

To further assess the subcellular localization of the 173 ranked candidates in each subcellular organelle in the heart, we utilized immunohistochemical data in the Human Protein Atlas^26^ to investigate their protein distributions and expression patterns in healthy human ventricular tissues (Fig. 4). Specifically, relative protein expression was obtained through the reported human heart muscle immunohistochemical images available at the Tissue Atlas. For these studies, we selected the highest ranked proteins in each subcellular organelle classification (Supplemental Table 1) and, where available, obtained human immunohistochemical images from the HPA. Using this approach, as expected COX20 and FAM162A showed localization to mitochondria of cardiomyocytes with strong mitochondrial expression patterns throughout the sections. Similarly, TMEM65, MCT1, SLC12A7, TLN2, MSN, FLOT1, SNTA1, DYSF, and AOC3 showed localization to the membranes of cardiomyocytes with particularly strong expression at cell surface and the cardiac intercalated disks. Immunohistochemistry analysis of REEP5 showed moderate expression and localization to the ER throughout the cells. Furthermore, STOM and CTNND1 revealed localization to the cytosolic space of cardiomyocytes. Similarly, LAMA2, LAMB2, and BGN showed subcellular localization throughout the cells perhaps in the protein secretory pathway. Immunohistochemistry analysis of MYH7, TNNT2, and TNNI3 were included as positive controls and demonstrated very strong, sarcomeric expression patterns and localization to cardiac muscle fibers in these healthy human ventricular tissues (Fig. 4).

**Fig. 5:**
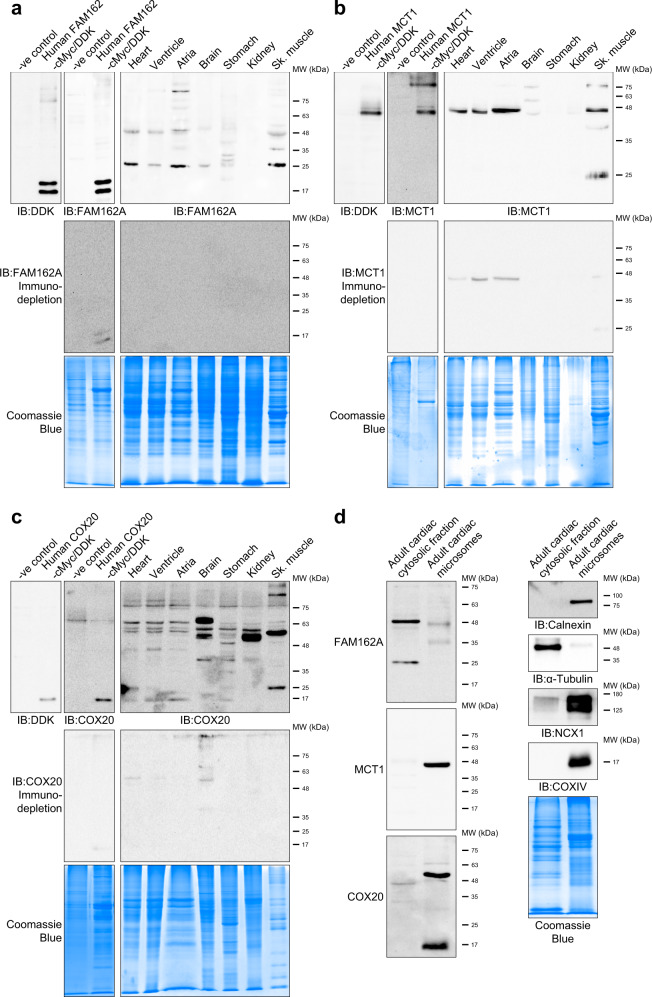
Immunblot analysis of protein expression across multiple tissues and microsomal fractions. Immunoblot analysis of (**a**) FAM162A, (**b**) MCT1, and (**c**) COX20 protein expression levels in mouse tissues. Blots were incubated with either anti-DDK antibody or FAM162A, MCT1 or COX20. Immunodepletion experiments were carried out by incubating primary antibodies together with HEK overexpression lysates. Coomassie blue gels were performed to visualize protein loading across all samples. (**d**) Immunoblot analysis of FAM162A, MCT1, and COX20 in isolated adult cardiac cytosolic fraction and enriched membrane microsomes from 10 mouse hearts. Blots were probed with primary antibodies as indicated, and in parallel, samples were run on a Coomassie blue gel. All immunoblots shown are representative of 3 total immunoblots performed, n = 3 independent biological replicates. Original uncropped immunoblots are provided in Supplemental Fig. 9. All original uncropped images were uploaded to figshare (10.6084/m9.figshare.11844972.v12).

As evidence of the utility of this refined dataset, we selected several highly ranked cardiac-enriched membrane-associated proteins to determine their protein expression across mouse tissues including FAM162A, MCT1, and COX20. We selected FAM162A, MCT1 and COX20 as prioritized candidates of novel proteins based on their highly muscle-specific tissue profiles along with MGI phenotypic analysis, representing the next logical targets for detailed follow-up biomolecular studies. In addition to the phenotypic analysis using the mouse genome informatics data, no known human mutations of FAM162A, MCT1, and COX20 have been linked to classical human heart diseases. To corroborate previous mRNA tissue expression analysis of FAM162A, MCT1, and COX20, immunoblot analysis of diverse adult mouse tissues (whole heart, ventricle, atria, whole brain, stomach, kidney, skeletal muscle) demonstrated strong protein expression in the whole heart in the heart for FAM162A (Fig. 5a), MCT1 (Fig. 5b), and COX20 (Fig. 5c). Notably, while FAM162A, MCT1, and COX20 demonstrated ventricular muscle enrichment, abundant expression of MCT1 was also detected in the atria and skeletal muscle (Fig. 5b). To examine antibody specificity against FAM162A, MCT1, and COX20, HEK overexpressed cMyc/DDK-tagged human FAM162A, MCT1, and COX20 purified lysates were obtained (Fig. 5a–c, left panels) for subsequent immuno-competition assays. For these experiments regarding FAM162A, immunoblot analysis of the HEK overexpressed human FAM162A lysate showed a clear doublet of FAM162A ~17 and 20 kDa. However, in the mouse tissues, higher molecular weight species of FAM162A were observed ~25, 35 and 48 kDa (Fig. 5a); although the implications of the potential oligomeric/altered status of FAM162A remains to be elucidated. Nonetheless, a clear enrichment of FAM162A antibody reactivity in striated muscle in the heart and skeletal muscle was apparent. Importantly, immunodepletion of the bands in the immunoblots using the FAM162A overexpression lysate demonstrated reduced intensity of bands observed at 17, 25, 35 and 48 kDa across all tissues indicating some degree of specificity of the reactivity (Fig. 5a, middle panel). MCT1 showed much cleaner antibody reactivity with a clear single band at ~42 kDa (predicted size) across nearly all tissues (Fig. 5b, top panels) that showed immunodepletion using purified cMyc/DDK-tagged MCT1 lysate, as the 42 kDa bands showed significant reduction in intensity (Fig. 5b, middle panel). The COX20 reactivity showed multiple band reactivity at 13 (predicted correct size), 25, 50, 58, 60 and 65 kDa (Fig. 5c, top panels). Immunodepletion using purified cMyc/DDK-tagged COX20 lysate was successful, as the multitude of bands visualized showed significant reduction in intensity under the competition conditions (Fig. 5c, middle panel). Lastly, immunoblot analysis of FAM162A, MCT1, and COX20 in isolated cardiac cytosolic fraction and microsomes showed a clear enrichment of MCT1 and COX20 in enriched membrane fractions (microsomes), along with the presence of FAM162A (Fig. 5d, left panels). While COX20 and MCT1 were not detected in the cytosolic fraction, FAM162A showed abundant expression also in this fraction. Control immunoblots were performed using calnexin (ER-enriched membrane protein enriched in microsomes), α-tubulin (cytosolic protein), sodium-calcium exchanger (NCX1; plasma membrane protein), and COXIV (inner mitochondrial membrane protein) (Fig. 5d, right panels) demonstrated successful subcellular fractionation of cardiac cytosolic and membrane fractions.

Given the identification of all three of these proteins in both human and mouse cardiomyocyte membrane proteomic isolations, we performed a detailed multi-species amino acid sequence analysis of FAM162A, MCT1, and COX20, with each of the three candidates exhibited a high degree of conservation throughout eukaryotic evolution. Specifically, FAM162A showed 79% homology between human and mouse FAM162A and 65% peptide conservation throughout evolution (Supplemental Fig. 5a). FAM162 is a family of proteins consisting two members, FAM162A and FAM162B. Our phylogenetic analysis demonstrated two distinct clustering of mammalian FAM162, demonstrating two diverse evolutionary descents within the FAM162 family (Supplemental Fig. 5b). Our RNA-Seq analysis of the FAM162 family further demonstrated that FAM162A shows dominant expression across various tissues in the FAM162 family (Supplemental Fig. 5c).

MCT1 exhibited 86% homology between human and mouse MCT1 and 78% peptide conservation throughout evolution (Supplemental Fig. 6a). MCT1 belongs to proton-linked monocarboxylate transporters consisting of a family of 14 members within the MCT family of proteins as demonstrated by our phylogenetic analysis yielding 14 distinct clustering of mammalian taxa (Supplemental Fig. 7a). While the expression distribution of MCT isoforms has been shown to be organism and tissue specific, our RNA-Seq analysis of various tissues showed that MCT1 is the predominant MCT in both the fetal and adult heart (Supplemental Fig. 7b)^27^.

Lastly, from zebrafish to mouse to monkey to human, COX20 demonstrated 81% homology between human and mouse COX20 and 69% peptide conservation throughout eukaryotic evolution (Supplemental Fig. 5d).

### FAM162A, MCT1, and COX20 transmembrane sequence prediction and membrane topology

**Fig. 6:**
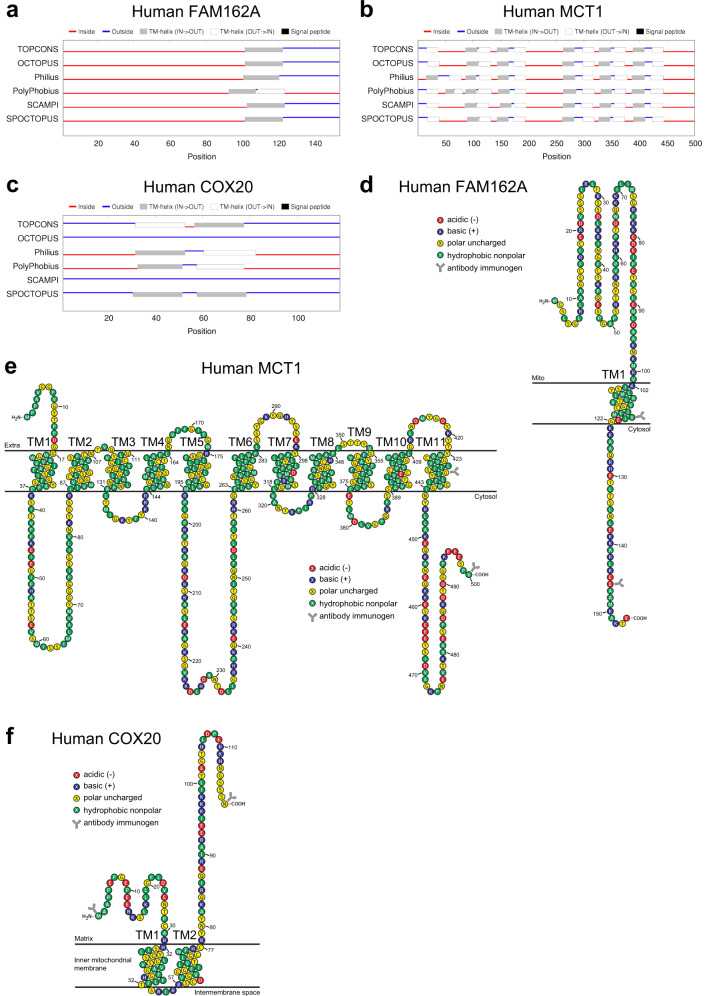
Membrane topology and prediction analysis of FAM162A, MCT1, and COX20. (**a**–**c**) Prediction of human FAM162A, MCT1, and COX20 protein topography generated by TOPCONS (http://topcons.cbr.su.se). (**d**–**f**) Predicted membrane topology model of human FAM162A, MCT1, and COX20A generated by modification of a T(E)Xtopo output. Peptide immunogen sequences for commercially available FAM162A, MCT1, and COX20 antibodies used in the current study are indicated by an antibody icon.

We explored human membrane topology using a multi-algorithm prediction tool (TOPCONS) based on available peptide sequences. We showed that a consensus FAM162A membrane topology model exhibited one transmembrane domain region with the N-terminus region residing in the inter mitochondrial membrane space and the C-terminus in the cytosol (Fig. 6a,d). Membrane topology and hydrophobicity prediction analyses of human MCT1 have proposed a structure with eleven transmembrane domains with the N-terminus in the extracellular space and the C-terminus in the cytosol as well as a large internal cytoplasmic loop between TM5 and TM6 (Fig. 6b,e). This prediction is consistent with the knowledge that MCTs possess ten to twelve transmembrane domains. However, structural labeling and proteolytic digestion studies of rat MCT1 has been shown to possess a total of twelve transmembrane domains with both N- and C-termini within the cytosol^28^. Lastly, our consensus membrane topology analysis of human COX20 confirmed two transmembrane domains with the hydrophilic domains of COX20 in the mitochondrial intermembrane space (Fig. 6c,f), consistent with the literature.

### FAM162A, MCT1, and COX20 endogenous expression and localization in cardiac myocytes

**Fig. 7:**
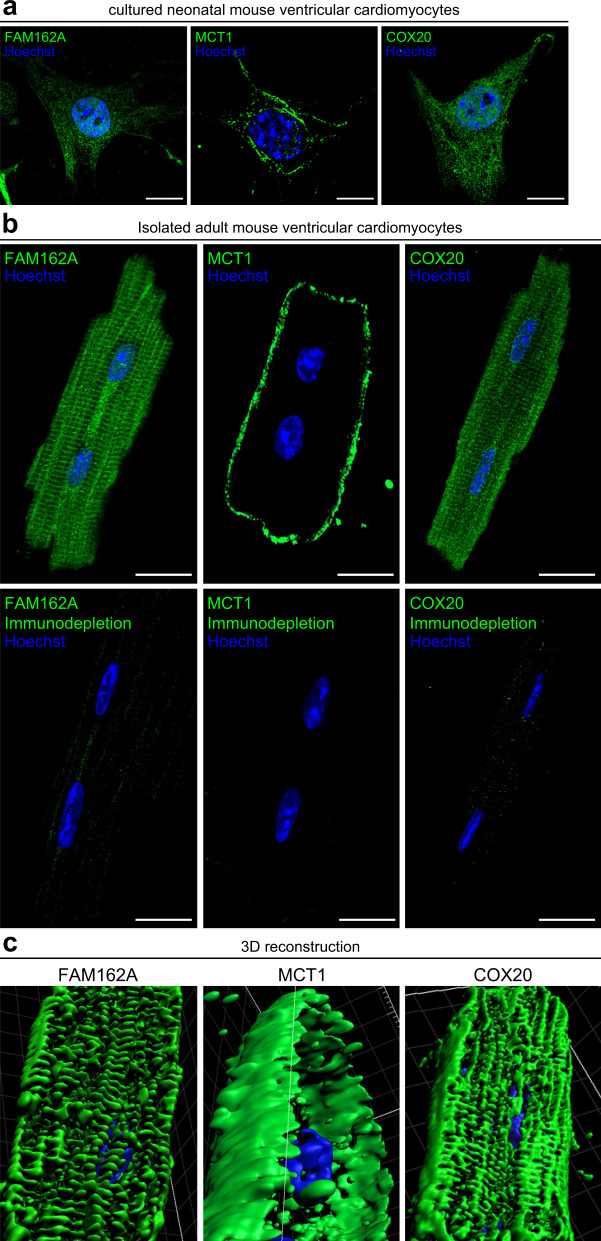
Immunofluorescence analysis of FAM162A, MCT1, and COX20 expression in mouse neonatal and adult ventricular cardiomyocytes. (**a**) Immunofluorescence analysis of endogenous FAM162A, MCT1, and COX20 expression (green) in isolated mouse neonatal ventricular cardiomyocytes. Nuclear staining was visualized with Hoechst staining (blue). Scale, 10μm. (**b**) Confocal imaging of endogenous FAM162A, MCT1, and COX20 expression (green) in isolated adult mouse ventricular cardiomyocytes demonstrate striated mitochondrial staining patterns for FAM162A and COX20, and exclusive plasma membrane localization of MCT1. Lower panels, imaging experiments were carried out by incubating reaction mixes of the primary antibodies with their respective overexpression protein lysates prior to incubating overnight. Scale, 20μm. (**c**) Three-dimensional reconstructive analysis using Imaris (Oxford Instruments) highlights the endogenous expression patterns of FAM162A, MCT1, and COX20. All images shown are representative of approximately 30–40 total images captured per condition, n = 3 independent biological replicates. All original uncropped microscopy images were uploaded to figshare (10.6084/m9.figshare.11844972.v12).

We next performed immunofluorescence analysis of endogenous expression pattern in primary cultured neonatal mouse ventricular cardiomyocytes and isolated adult mouse ventricular cardiomyocytes for FAM162A, MCT1, and COX20. FAM162A demonstrated typical mitochondrial staining pattern in cultured neonatal cardiomyocytes (Fig. 7a) and a very organized mitochondrial staining pattern in isolated adult mouse cardiomyocytes (Fig. 7b). MCT1 showed clear localization to the plasma membrane in cultured neonatal mouse ventricular cardiomyocytes and isolated adult mouse cardiomyocytes (Fig. 7a,b). Endogenous COX20 expression exhibited characteristic mitochondrial staining patterns in cultured neonatal mouse ventricular cardiomyocytes and isolated adult mouse cardiomyocytes (Fig. 7a,b) similar to FAM162A patterns. To validate the specificity of the staining patterns, our immunodepletion assays using cMyc/DDK-tagged FAM162A, MCT1, and COX20 lysates successfully masked the specific signals shown in Fig. 7b top panels in cardiomyocytes. Additionally, three-dimensional reconstructive analysis of our confocal data was performed in the isolated adult cardiomyocytes for all three proteins. FAM162A and COX20 showed longitudinal cardiac mitochondria as well as localization between rows of myofibrils (Fig. 7c). Three-dimensional reconstructive analysis of endogenous MCT1 expression in isolated adult mouse cardiomyocytes showed strong expression in the plasma membrane and cardiac intercalated disks (Fig. 7c).

**Fig. 8:**
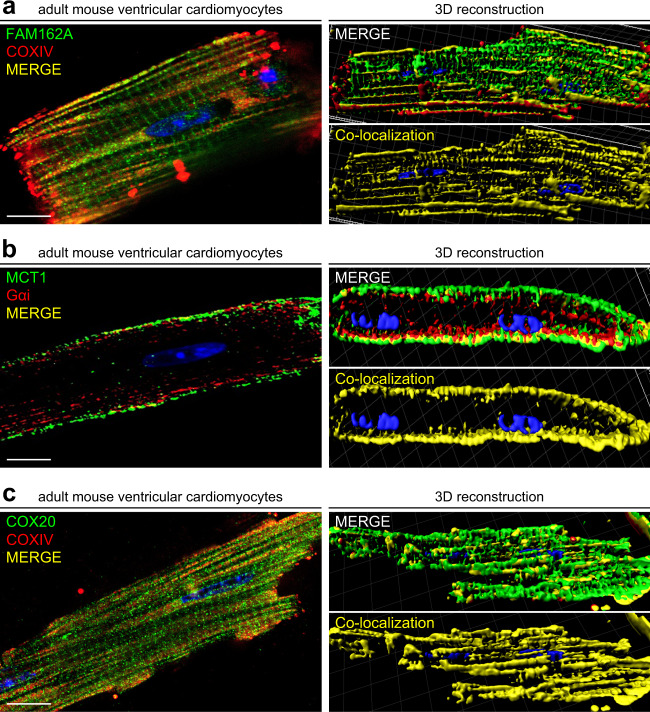
Co-immunofluorescence analysis demonstrates colocalization of FAM162A and COX20 with known mitochondrial marker, COXIV, and MCT1 colocalization with known plasma membrane protein, Gαi, in isolated adult mouse cardiomyocytes. (**a**) Immunofluorescence analysis of FAM162A (green) co-stained with mitochondrial protein, COXIV (red) in acutely isolated adult mouse cardiomyocytes. Three-dimensional reconstructive analysis demonstrates regions of colocalization (yellow) with a Pearson’s coefficient p > 0.5. Scale, 10 μm. (**b**) Immunofluorescence analysis of MCT1 (green) co-stained with known plasma membrane protein, Gαi (red) in acutely isolated adult mouse cardiomyocytes. Three-dimensional reconstructive analysis demonstrates regions of colocalization (yellow) with a Pearson’s coefficient p > 0.5. Scale, 10μm. (**c**) Immunofluorescence analysis of COX20 (green) co-stained with mitochondrial protein, COXIV (red) in acutely isolated adult mouse cardiomyocytes. Three-dimensional reconstructive analysis demonstrates regions of colocalization (yellow) with a Pearson’s coefficient p > 0.5. Scale, 10μm. Nuclear staining was visualized with Hoechst staining (blue). All images shown are representative of approximately 30–40 total images captured per condition, n = 3 independent biological replicates. All original uncropped microscopy images were uploaded to figshare (10.6084/m9.figshare.11844972.v12).

To further corroborate the specificity of the staining patterns, we performed immunofluorescence co-staining analysis of FAM162A with COX IV, a known mitochondrial membrane marker, and demonstrated a high degree of co-localization in the mitochondria in isolated adult mouse cardiomyocytes (Fig. 8a, left panels and Supplemental Fig. 8a). Three-dimensional reconstructive analysis confirmed significant, strong co-localization of FAM162A (Pearson coefficient of 0.76 ± 0.16; *p* < 0.05) with COX IV in adult cardiomyocytes (Fig. 8a, right panels). Next, we compared the expression of MCT1 with that of a known plasma membrane protein, G protein inhibitory alpha subunit (Gαi) which demonstrated strong expression of both MCT1 and Gαi in their respective subdomains in the plasma membrane and MCT1 in the intercalated disks of cardiomyocytes (Fig. 8b and Supplemental Fig. 8b). Three-dimensional reconstructive analysis further demonstrated strong co-localization between MCT1 and Gαi at the plasma membrane (Pearson coefficient of 0.53 ± 0.03; *p* < 0.05) (Fig. 8b). Lastly, immunofluorescence analysis of COX20 co-stained with a mitochondrial marker, COX IV, demonstrated strong subcellular mitochondrial co-localization throughout the cell (Pearson coefficient of 0.61 ± 0.12; *p* < 0.05) (Fig. 8c and Supplemental Fig. 8c).

### Differential regulation of FAM162A, MCT1, and COX20 protein expression within the myocardium of heart failure patients

**Fig. 9:**
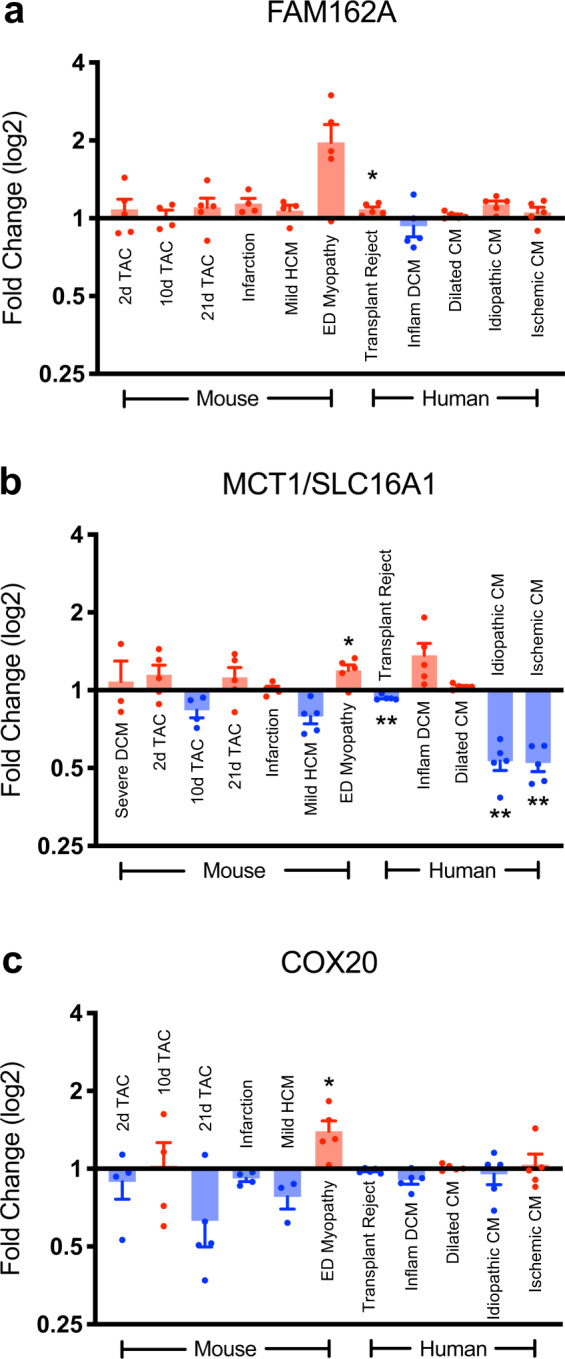
GEO transcriptomic analysis of FAM162A, MCT1, and COX20 mRNA transcript levels in various mouse and human heart diseases. (**a**–**c**) GEO RNA profiles demonstrate alterations in FAM162A, MCT1, and COX20 expression across various mouse and human heart diseases. Data are reported as normalized hybridization signals. Red bars indicate increased expression against normal hearts, blue bars indicate decreased expression against normal hearts, n = 3–5 available biologically independent measurements. Asterisks indicate a statistically significant *p* value in a Tukey’s multiple comparison analysis where *p < 0.05 and **p < 0.01; data are presented as mean ± SEM. All source data input and analyzed output files were uploaded to figshare (10.6084/m9.figshare.11844972.v12).

To assess for any correlation to cardiac disease, we performed a query of publicly available GEO RNA-Seq datasets containing data for human and mouse cardiovascular diseases to determine expression levels of FAM162A, MCT1, and COX20. Specifically, FAM162A transcript levels were upregulated in several mouse disease models including transverse aortic constriction-induced (TAC) heart failure, myocardial infarction, and hypertrophic cardiomyopathy (Fig. 9a). Consistent with the mouse data, an increased expression of FAM162A was observed in human dilated cardiomyopathy (DCM), idiopathic cardiomyopathy, and ischemic cardiomyopathy (ICM), albeit not significantly. Interestingly, MCT1 transcript levels changed uniquely in human heart diseases. While a relatively inconsistent trend of MCT1 expression levels was observed with several mouse disease models, a marked reduction in MCT1 transcript levels were detected in human idiopathic and ischemic cardiomyopathies, perhaps suggesting a link in ICM disease progression (Fig. 9b). Similarly, decreased levels of COX20 transcripts were observed in mouse TAC, infarcted, and hypertrophic hearts (Fig. 9c). In agreement with these data, human DCM and idiopathic cardiomyopathy also showed a downregulation of COX20 expression, albeit not significantly (Fig. 9c). However, we acknowledge that these data are observational in nature and do not establish a causal or direct link between the altered expression and cardiac dysfunction.

**Fig. 10:**
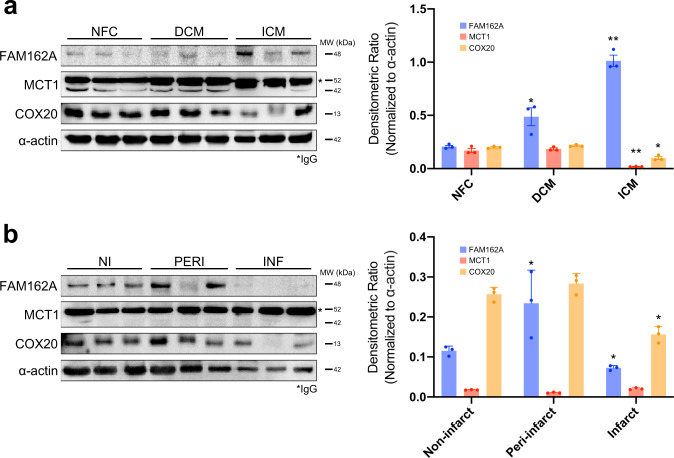
FAM162A, MCT1, and COX20 protein expression levels in human adult DCM and ICM heart failure patients. (**a**) Immunoblot analysis of FAM162A, MCT1, and COX20 in human adult DCM and ICM patient samples. Right panel, densitometric quantification of the blots. Significantly higher levels of FAM162A in human adult DCM and ICM patients, and significantly reduced levels of MCT1 and COX20 protein expression in ICM patients were measured. (**b**) Immunoblot analysis of FAM162A, MCT1, and COX20 in region-specific infarcted hearts. Right panel, densitometric quantification of the blots. All immunoblots shown are representative of at least 3 total immunoblots performed, n = 3 independent biological replicates. Original uncropped immunoblots are provided in Supplemental Fig. 9. Asterisks indicate a statistically significant *p* value in a Tukey’s multiple comparison analysis where *p < 0.05 and **p < 0.01; data are presented as mean ± SEM. All source data input and analyzed output files were uploaded to figshare (10.6084/m9.figshare.11844972.v12).

In an attempt to establish the link between altered expression and human heart diseases, we performed immunoblot analysis of FAM162A, MCT1, and COX20 to investigate protein levels in human hearts from patients with clinical DCM and ICM, the two most common etiologies of heart failure. Patient baseline data and clinical characteristics are reported in Table 1. We first compared their expression levels in human DCM and ICM myocardial tissues to non-failing myocardial controls (NFC). Protein levels of FAM162A were found significantly elevated in DCM and to a greater extent in ICM hearts; whereas MCT1 and COX20 expression levels were significantly reduced in ICM hearts only (Fig. 10a). A near complete depletion of MCT1 expression in ICM hearts, but not DCM hearts, showed apparent disease-specific changes in the expression levels of MCT1 and may provide functional insights to ICM-induced heart failure (Fig. 10a). MCT1 has been reported at 40–48 kDa in previous studies in human muscle tissues^29,30^, and consistent with the literature, we detected molecular bands of MCT1 at 42 kDa. The presence of a higher molecular weight band (~52 kDa) across all our human blots was suspected to be IgG. Next, assessment of protein expression in the distinct regions of the left ventricular myocardium of ICM patients was performed using myocardial tissues from infarct, peri-, and non-infarct regions of the left ventricle to explore intra-cardiac differences (Fig. 10b). Markedly elevated FAM162A protein expression levels were measured in the dynamic remodeling region of the peri-infarct relative, and levels were nearly absent in the infarct zone which is composed of fibrotic scar mostly (Fig. 10b). Furthermore, the relatively low MCT1 expression in ICM hearts compared to NFC and DCM hearts was shown to be consistent within the whole left ventricle of ICM hearts, whereas decreased COX20 protein levels appeared to be restricted to the infarct region (Fig. 10b). These data demonstrate that FAM162A upregulation occurred in both models of heart failure, and that MCT1 and COX20 decreased levels occurred in ICM, suggesting perhaps distinct roles for these protein candidates. However, two major limitations for consideration when evaluating human samples are the heterogeneous cell populations and inter-individual variability which may have resulted in altered expression ratios observed in our study. Future investigations with a larger patient sample size and follow-up biochemical studies in isolated cardiomyocytes are required to corroborate and extend these initial findings.

**Table 1:** Patient baseline and clinical characteristics.

Etiology	Patient code	Gender	Age at transplant (years)	EF (%)	Duration of HF (months)	Hb (g/L)	eGFR (mL/min/1.73 m^2^)	Medications (1 = yes; 0 = no)		
								ACEi/ARB	Beta-blocker	MRA
ICM	ICM1	M	55	22	73	129	59	1	1	1
	ICM2	M	62	24	64	130	54	1	1	1
	ICM3	F	51	19	58	123	73	1	1	1
DCM	DCM1	M	51	15	60	122	71	1	1	1
	DCM2	M	45	25	75	134	63	1	0	1
	DCM3	F	55	29	81	131	79	1	1	0
NFC	NFC1	F	42	—	—	—	—	—	—	—
	NFC2	M	38	—	—	—	—	—	—	—
	NFC3	M	54	—	—	—	—	—	—	—

EF, ejection fraction; Hb, hemoglobin; eGFR, estimated glomerular filtration rate; ACEi/ARB, angiotensin-converting enzyme inhibitor/angiotensin receptor blocker; MRA, mineralcorticoid receptor antagonist.

## Discussion

The process of cardiac contraction is precisely controlled by many essential membrane proteins to generate electrical signals and mechanical forces needed to pump the heart. For this reason, membrane biology is perhaps the most important, yet most difficult to study in cell biology due to poor solubility and relatively low expression of this class of protein. Here, we used quantitative mass spectrometry data, informatics and imaging analysis of human and mouse cardiomyocyte to investigate these cardiac-relevant, membrane proteins. We identified 173 membrane-associated proteins as a result of this analysis. We have provided further compelling evidence suggesting that some of our prioritized rank-ordered list of proteins, including FAM162A, MCT1, and COX20 are cardiac enriched, localized within subcellular compartments, and may play a role in the progression of heart failure.

While FAM162B has been linked to congenital colon disease as a result of lacking nerve cells in the colon^31^, FAM162A has been shown to function as a pro-apoptotic molecule involved in facilitating hypoxia-induced mitochondrial apoptosis^32,33^. Specifically, *in vitro* overexpression of FAM162A induces canonical mitochondrial cell death in prostatic cancer cells and human alveolar epithelial cells. More excitingly, FAM162A targeted suppression has been shown to attenuate apoptosis, demonstrating its anti-apoptosis potential for potential alternative therapeutic strategy^32,34^. However, the specific role and detailed subcellular localization of FAM162A in the myocyte in the heart has not been explored.

MCT1 is best known as a proton-coupled monocarboxylate transporter that mediates the transport of many monocarboxylates such as lactate, pyruvate, ketone bodies, and various amino acids across the plasma membrane. The regulation of these small molecules contributes to a multitude of metabolic pathways during normal physiological as well as pathological conditions. The molecular function of MCT1 and its importance for normal physiological metabolism have been characterized, previously^35^. However, considerably less information is available regarding the precise role of MCT1 in cardiac metabolism under normal and pathological conditions. In heart failure, a metabolic shift in energy substrate is established, favoring carbohydrate utilization as well as increased lactate transport to improve heart function in response to an insult^36^. In fact, increased MCT1 expression in a rat model of myocardial infarction and congestive heart failure has been observed, suggesting the critical initial compensatory role of MCT1 in transporting lactate as a metabolic resource in the failing heart^37^.

Finally, the assembly of cytochrome c oxidase in the mitochondria is clearly essential for mature cellular respiration and aerobic metabolism in bacteria and eukaryotes^38^. COX20, an inner mitochondrial membrane protein, has been shown to play an important role in organizing efficient cytochrome c oxidase assembly, functioning as a chaperone for the process^39^. Specifically, the hydrophilic domains of COX20 have been shown to be required for interactions with COX2 and COX18, two conserved subunits of the cytochrome c oxidase complex, to ensure successful assembly and respiratory growth^39^. Furthermore, mutations in human COX20 have been linked to loss of neurological control of muscle movements resulting in muscle hypotonia and dystrophy^40,41^. However, the phenotypic spectrum of COX20 expression alterations has not been investigated to date despite its high expression levels in the ventricle in the heart.

Elucidating the complete membrane proteome of cardiomyocytes furthers our understanding of myocyte biology in both the healthy and diseased heart. Our in-depth membrane proteomic analyses followed by *in vitro* validation in functional adult mouse cardiomyocytes as well as detailed immunoblot analysis from human heart biopsies have provided compelling evidence and suggested a link between these previously uncharacterized, cardiac-enriched membrane proteins to heart disease progression in the human heart. However, it is important to acknowledge that abundance may not be a sufficient indicator of translational significance in function. In this study, we have prioritized candidate proteins that show cardiac enrichment as a first step in characterizing previously understudied cardiac membrane-associate proteins. The role and contribution of many of the non-cardiac-enriched candidate proteins, with no previous reported cardiac phenotype shown in Supp. 3, will likely be clarified with detailed future studies.

Furthermore, we have characterized their subcellular localization and expression patterns in the myocyte and performed detailed, region-specific protein expression analysis in dilated cardiomyopathy and ischemic cardiomyopathy. Detailed comparison of the dynamic regional subgroups, such as non-infarct, peri-infarct, and infarct regions of the ischemic hearts provides key insights into understanding the basis and transition between healthy and diseased myocardium. A marked increase in FAM162A expression level in the peri-infarct regions of the infarcted hearts suggest its role in the progression of ICM; we speculate where it may be involved in inducing mitochondrial apoptosis and thus establishing the pathological phenotypes in response to ischemic insults in the heart. Similarly, an upregulation of FAM162A in human DCM hearts suggests its specific role in modulating the etiologies of this specific heart disease characterized by dilated and impaired ventricular contraction. Defects in mitochondrial proteins in the heart are a frequent cause of cardiac myopathies as cardiomyocytes are mitochondria enriched and the myocyte relies on the mitochondria to generate sufficient ATP to fuel beat-to-beat contractions. Future studies investigating the therapeutic potential of FAM162A depletion in the failing heart would thus be invaluable. Moreover, a near complete deletion of MCT1 expression in infarcted hearts- irrespective of ventricular region- may indicate a crucial importance in maintaining normal cardiac physiology and its direct contribution to ICM. However, the specific mechanisms will require ongoing investigations, with future conditional, tissue-specific knockout studies in the heart providing immeasurable insights and quality control into their specific roles in regulating heart function. Until then, ongoing work will continue refine the role of FAM162A, MCT1, and COX20 in the heart.

Lastly, it is important to acknowledge that this study is primarily explorative and observational in its nature. Obviously, future studies must focus on the precise molecular functions of these identified proteins in cardiac health and disease to determine their specific role(s) in regulating heart development and/or function. Moreover, although we attempted necessary controls where possible, antibody-based experiments should always be assessed and interpreted very carefully. The antibodies used in this study were carefully selected, with previous validations for specificity, however, accuracy of these subcellular localization data of FAM162A, MCT1, and COX20 in cardiomyocytes depend largely on the quality of the primary antibodies used in our confocal studies. Despite such limitations, our co-immunofluorescence studies with known mitochondrial and plasma membrane markers and consistent data from three-dimensional reconstructive imaging analysis along with high sample numbers were designed to minimize any potential confounding factors in these studies.

Taken together, our study represents an integrated analysis of cardiomyocyte cell surface proteome to elucidate promising novel membrane candidates of heart function and potential therapeutic targets for heart disease. Our human and mouse cardiomyocyte membrane proteomes can be used as a reference for future studies aiming to identify and characterize heart disease markers and therapeutic targets for heart failure. Future deep membrane proteomics studies of cardiomyocytes with combined proteomic investigations of cardiac membrane protein PTM (post-translational modifications) will help complete the membrane proteome of the human heart and provide molecular mechanistic insights into the various disease etiologies of heart failure.

## Methods

Cell-surface membrane proteome and identification of previously uncharacterized, evolutionarily conserved, cardiac-enriched membrane proteins. Previously, we enriched for membrane fractions from mouse neonatal ventricular cardiomyocytes and human fetal cardiomyocytes using cationic colloidal silica beads^10,11^. In those experiments, a membrane-depleted homogenate (removed after centrifugation at 1000 g for 5 min, labelled ‘H’) as well as a membrane-enriched fraction (pellet resuspended in NET buffer (400 mM NaCl, 25 mM Hepes pH7.4, and 1% Triton-X100) for 2 hours at 4 °C). were then subjected MudPIT^42^ (Multidimensional Protein Identification Technology) analysis with stringent statistical filtering (<1% false discovery rate) as previously described^10^. This analysis resulted in the identification of 2,762 human and 3,033 mouse proteins. The original raw mass spectrometry files were searched using X!TANDEM (version 2010.12.01.1; ftp://ftp.thegpm.org/projects/tandem/source/2010-12-01/) against human or mouse UniProt database (version 2011_03; ftp://ftp.uniprot.org/pub/databases/uniprot/previous_releases/release-2011_01/) with the following search parameters: Parent ion Δ-mass of 4 Da, fragment mass error of 0.4 Da, and complete carbaminomethyl modification of cysteine by iodoacetic acid. The resulting tryptic peptides matching these criteria made up the final list of identified proteins. Next, to identify conserved membrane-associated proteins, orthology mapping analysis of all identified human and mouse proteins was performed using the MGI orthology database (http://www.informatics.jax.org) taking into account only one-to-one orthology to yield the identification of human and mouse one-to-one orthologues^43^. Comparative proteomics analysis using Qspec identified proteins that were enriched in the TX-100 fraction compared to the homogenate fraction, resulting in the identification of the membrane-enriched orthologue proteins (Supplementary Table 1).

The identified 555 proteins were converted to 550 as 5 IDs (TFRC_human P02786, mouse Q62351; TIMM50_human Q3ZCQ8, mouse Q9D880; PSPC1_human Q8WXF1, mouse Q8R326; GNAS_human P84996, mouse Q6R0H6; TMPO_humanP42167, mouse Q61029) were amalgamated into unique IDs. To rank the 550 membrane-enriched proteins identified, we first carried out prediction of transmembrane domains of the 550 identified proteins using TMHMM version 2 (http://www.cbs.dtu.dk/services/TMHMM) to identify membrane localized protein clusters. To further select novel cardiac membrane-enriched proteins, MGI phenotypic analysis (http://www.informatics.jax.org/phenotypes.shtml) was performed to individually map against our datasets to select and rank novel cardiac membrane proteins. Finally, we manually annotated the 550 protein clusters to select cardiac enriched transcripts by employing the publicly available microarray database, BioGPS microarray data (http://biogps.org/) to complete the rank ordered analysis of the 550 identified proteins. Excel output file containing full ranking criteria along with step-by-step ranking of the 550 identified proteins (labelled as “Ranking Criteria + Step-by-step ranking) was uploaded to figshare (10.6084/m9.figshare.11844972.v12).

### Human myocardium procurement

Cardiac tissue samples were collected from donors with no history of heart disease and patients with dilated cardiomyopathy or ischemic heart disease during their cardiac transplantation through the HOPE program (Human Organ Procurement and Exchange program, University of Alberta, Edmonton, Canada) and the Human Explanted Heart Program (HELP) respectively under the approval of the Human Research Ethics Board at the University of Alberta^44,45^. Specifically, following cold cardioplegia, myocardial samples were excised from the left ventricular anterior free wall and immediately flash-frozen to be stored at −140 °C. Clinical review of 12-lead ECG and coronary angiogram were used to identify the infarct, peri-, and non-infarct regions of the left ventricle in ischemic heart disease patients. Patient samples from each category (Non-failing control; n = 3, ICM; n = 3, DCM; n = 3) were collected and used in this study. Patient baseline and clinical characteristics including age, gender, duration of heart failure, ejection fraction, hemoglobin levels, eGFR, and medication history are presented in Table 1.

### Adult and neonatal mouse cardiomyocyte isolation

All experimental procedures involving animals were approved by the University of Toronto Animal Use and Care Committee; all animal experiments were conducted in accordance with the animal care guidelines. Adult mouse ventricular cardiomyocytes were isolated from adult male 6–8 week old CD1 mice (Jackson Labs)^46,47^. The outbred CD1 mouse strain was used due to their genetic diversity, larger litter size, and we have used these stains in our ongoing research programs where we have refined cardiomyocyte isolation from CD1 mouse hearts for *in vitro* genetic and small-molecule manipulation in our lab^47^. Briefly, mice were euthanized with isoflurane and hearts perfused with EDTA buffer containing 15 μmol/L of blebbistatin with the heart clamped at the ascending aorta, followed by tissue digestion with collagenase type II (525 units/mL; Worthington Biochemical Corp.). The isolate was then filtered through a 70μm strainer and gravity-settled for 15 min to allow rod-shaped cardiomyocytes to settle by gravity and form a pellet. Dissociated ventricular cardiomyocytes were seeded on Geltrex-coated glass-bottom dishes for subsequent experiments. Primary neonatal mouse ventricular cardiomyocytes were isolated and cultured from 1–3 day old CD1 pups as described previously^46^. Isolated ventricles were enzymatically digested in 0.05% trypsin overnight at 4 °C, followed by collagenase type II digestion (450 units/mL) at 37°C. Collected ventricular cardiomyocytes were seeded and maintained on gelatin-coated glass-bottom dishes for 48 hours prior to any experiments.

### Publicly available RNASeq and proteomic datasets

Publicly available data sources of mRNA Affymetrix transcript data from the GEO Profiles in NCBI including several mouse and human heart diseases were downloaded and analyzed. FAM162A: 2d, 10d, 21d TAC (GPL81, 160218_at), MI (GPL5371, 20160), alpha-tropomyosin mild HCM (GPL339, 1451385_at), Emery-Dreifuss cardiomyopathy (GPL1261, 1443339_at), heart transplant (GPL96, 220942_x_at), Inflammatory DCM (GPL570, 220942_x_at), DCM (GPL6244, 8082066), and idiopathic and ischemic cardiomyopathies (GPL570, 220942_x_at); MCT1/SLC16A1: Severe DCM (GPL32, 101588_at), 2d, 10d, 21d TAC (GPL81, 101588_at), MI (GPL5371, 12952), alpha-tropomyosin mild HCM (GPL339, 1415802), Emery-Dreifuss (GPL1261, 1415802), heart transplant (GPL96, 202235), Inflammatory DCM (GPL570, 202235_at), DCM (GPL6244, 7918622), and idiopathic and ischemic cardiomyopathies (GPL570, 1557918_s_at); COX20: 2d, 10d, 21d TAC (GLP81, 160315_at), MI (GPL5371, 1309), alpha-tropomyosin mild HCM (GPL339, 1428619_at), Emery-Dreifuss (GPL1261, 1428619_at), heart transplant (GPL96, 206848_at), Inflammatory DCM (GPL570, 224820_at), DCM (GPL6244, 7911085), and idiopathic and ischemic cardiomyopathies (GPL570, 224820_at). Data were reported as normalized hybridization signals. Comprehensive RNA-Seq based transcript levels of multiple mouse organs were obtained from the Human Protein Atlas^26^. Data were reported as the abundance in ‘Transcripts per Million’ (TPM) in the heart, relative to the sum of the TPM values across all tissues^26^. All source data input and analyzed output files (labelled as “Fig. 3_Cardiac-enriched + noMGI”, “Fig. 9 GEO profiles raw data”, “Supp. 1_HPM raw data”, “Supp. 2_Cardiac-enriched + MGI”, “Supp. 3_Noncardiac-enriched + noMGI”, “Supp. 4_Noncardiac-enriched + MGI”, “Supp. 5_HPM raw data”, and “Supp. 7_HPM raw data”) were uploaded to figshare (10.6084/m9.figshare.11844972.v12).

To assess the subcellular localization in subcellular organelles in the heart, we utilized immunohistochemical data available in the Human Protein Atlas^26^ to investigate their protein distributions and expression patterns in healthy human ventricular tissues. Images for proteins were downloaded from the Atlas which contains images of histological sections from normal and cancer tissues. Specific antibodies were labeled with DAB (3,3’-diaminobenzidine) with a resulting brown staining indicative of antibody reactivity, with counterstained hematoxylin to enable visualization of nuclei. Images were all available at https://www.proteinatlas.org/humanproteome/tissue/heart^26^, and images used in Fig. 4 were uploaded (labelled as “Fig. 4_HPA original uncropped images”) to figshare (10.6084/m9.figshare.11844972.v12).

### Antibodies and reagents

Primary rabbit polyclonal anti**-**FAM162A antibody (PA5-24470, ThermoFisher; IB: 1:1000 dilution, IF: 1:800 dilution), rabbit polyclonal anti-COX20 antibody (25752-I-AP, Proteintech; IB: 1:1000 dilution, IF: 1:200 dilution), mouse monoclonal anti-MCT1 antibody (MA5-18288, ThermoFisher; IB: 1:1000 dilution, IF: 1:500 dilution) and rabbit polyclonal anti-MCT1 antibody (PA5-72957; ThermoFisher; IB: 1:1000 dilution, IF: 1:500 dilution) were used for immunofluorescence and immunoblot studies. These antibodies were carefully selected where previous validation data have been reported. Specifically, the MCT1 antibody was validated in CRISPR/Case9 siRNA-mediated knockdown HEK293 cells where a complete depletion of MCT1 expression was observed in immunoblot analysis (ThermoFisher, P14612), the COX20 antibody was validated in immunohistochemistry and immunofluorescence studies of various human tissues and cell lines (Proteintech, 25752-1-AP), and lastly, the FAM162A antibody was validated in immunoblot experiments where a clear protein band was detected at the expected molecular weight with minimal nonspecific bands (ThermoFisher, PA5-24470). The following immunogen sequences for the abovementioned commercially available antibodies are indicated in Fig. 6d–f: peptide sequence between 118–146 amino acids from the C-terminal region of human FAM162A; peptide fragment within amino acids 436–500 of human MCT1; peptide sequence corresponding to amino acids 1–118 amino acids of human COX20. Additionally, primary mouse monoclonal anti-COX IV antibody (ab14744, abcam; IF: 1:1000 dilution) and rabbit polyclonal anti-Gαi antibody (#21006, NewEast Biosciences; IF: 1:800 dilution) were used for immunofluorescence co-staining studies. Lastly, mouse anti-alpha actin antibody (JLA20, DSHB) was used for immunoblot studies at 1:1000 dilution.

Similarly, antibody sensitivity and specificity are of essential importance in ensuring correct interpretations for expression patterns in healthy human myocardial tissues presented in Fig. 4a. All antibodies used for the presented immunohistochemistry data were validated previously where a unique protein epitope signature tag with high specificity was selected for each antibody used in immunohistochemistry studies followed by immunoblot analysis from limited tissues (liver, tonsil) and cell lines (RT4, U-251 MG). Finally, the observed staining pattern was assigned a reliability score based on concordance with experimental protein characterization in the UniProt database^48,49^. In addition to the standard antibody validation procedures, enhanced antibody validation was performed for MCT1, TLN2, MSN, FLOT1, DYSF, AOC3, CTNND1, LAMA2, BGN, MYH7, TNNT2, and TNNI3 antibodies where genetic and orthogonal validations were performed to confirm antibody specificity for immunohistochemistry assays.

### Immunoblot analysis

Protein lysates from mouse tissues were harvested in radioimmunoprecipitation assay buffer (RIPA) (50 mM Tris-HCl; pH7.4, 1% NP-40, 0.5% sodium deoxycholate, 0.1% SDS, 150 mM NaCl, and 2 mM EDTA), supplemented with EDTA-free protease inhibitor cocktail (Roche), for 30 minutes on ice, spun down at 15,000 × *g* at 4 **°**C as previously described^50^. Human myocardium samples were harvested in 8 M urea supplemented with protease and phosphatase inhibitor cocktails (Roche) and spun down at 15,000x*g* at 4**°**C for 15 minutes to remove tissue debris. Soluble fractions (supernatant) were then saved for immunoblotting studies. Standard SDS-PAGE chemiluminescent analysis was performed and tissue lysates were run on 10–12% polyacrylamide gels and transferred onto 0.45μm nitrocellulose membranes. Primary antibodies were added after blocking with 5% milk in 0.05% TBS-Tween20 for 1 hour and incubated at 4**°**C overnight. Secondary antibodies of anti-mouse (W402B, Promega) and anti-rabbit (W401B, Promega) HRP-conjugated IgG were used to at 1:2500 dilution. Original uncropped immunoblots are available and provided as Supplemental Fig. 9. Immunodepletion experiments were performed by incubating primary antibodies with 10 μg of purified cMyc/DDK-tagged human FAM162A (Origene, LY415331), cMyc/DDK-tagged human MCT1 (Origene, LY401069), or cMyc/DDK-tagged human COX20 (Origene, LY405090) lysates in 10 mL of 5% milk (1μg /mL) for 3 hr at 4**°**C prior to probing the membrane at 4**°**C overnight.

### Immunofluorescence and confocal microscopy

Acutely isolated adult mouse ventricular cardiomyocytes and cultured neonatal mouse ventricular cardiomyocytes were seeded on imaging glass-bottom dishes (MatTek, MA, USA) for immunofluorescence analysis. All immunofluorescence procedures were performed as described previously^46^. Briefly, cardiomyocytes were fixed with 4% paraformaldehyde for 30 minutes for adult cardiomyocytes and 15 minutes for neonatal cardiomyocytes on ice followed by permeabilization with 0.5% Triton X-100, 0.2% Tween-20, and 5% FBS in PBS) for 30 minutes at room temperature. Cardiomyocytes were then incubated with primary antibodies in permeabilization overnight at 4**°**C. Samples were then incubated with fluorophore-conjugated secondary antibodies the next day (Alexa Fluor 488 or Alexa Fluor 647, Molecular Probes) at 1:1000 dilution. Samples were visualized using a Zeiss spinning-disk confocal microscope. Immunodepletion experiments were performed by incubating primary antibodies with 2 μg of purified cMyc/DDK-tagged human FAM162A (Origene, LY415331), cMyc/DDK-tagged human MCT1 (Origene, LY401069), or cMyc/DDK-tagged human COX20 (Origene, LY405090) lysates in 1 mL of permeabilization buffer for 3 hr at 4**°**C prior to incubating cardiomyocytes with primary antibodies at 4**°**C overnight.

### Image processing and analysis

Image processing and analysis of acquired images were performed using the Zeiss ZEN software (ZEN 2012 blue edition). Specifically, the unsharp masking tool was applied to all exported images to enhance subtler structures using ‘auto-thresholding’. All three-dimensional reconstructive analysis of z-stack images were performed using Imaris 8.1 (Bitplane, Switzerland). Three-dimensional reconstruction and co-localization models were built using surpass surface reconstruction, an intensity-based rendering of three-dimensional volume of z-stack images with ‘auto-thresholding’ set at Pearson’s coefficient p > 0.5.

### Experimental design and statistical rationale

Searched mass spectrometry data was downloaded from Sharma *et al*.^10^, whereby three independent biological replicates including three technical replicates within each biological replicate were used for further informatic analyses. For GEO RNA-Seq and immunoblot studies, statistical analyses were analyzed using the GraphPad Prism 8 software. Descriptive statistics are reported and shown as mean ± SEM. All bar graphs showing individual data points with error bars are graphically presented using the GraphPad Prism 8 software. Normally distributed data were analyzed using one-way ANOVA followed by *post hoc* Tukey’s multiple comparison test for each mean comparison. Experimental mean-fold protein intensities, relative to controls from triplicate assays, were considered different from controls at the *p* ≤ 0.05 significance level. For visual representation, a *p* value ≤ 0.05 is denoted by *; a *p* value ≤ 0.01 is denoted by **, and a *p* value ≤ 0.001 is denoted by ***. All immunoblots shown are representative from a minimum of three independent biological replicates; all original uncropped immunoblots are provided in Supplemental Fig. 9. All data figures and tables as well as all supporting information were uploaded to figshare (10.6084/m9.figshare.11844972.v12).

## Supplementary information

Supplemental Table 1

Supplementary Information

## Data Availability

All data records and supporting materials have been deposited in proteomeXchange and figshare. Specifically, MudPIT excel output files obtained from the Supplementary Table files of Sharma *et al*.^10^ containing original unique peptides and total spectra detected files (labelled as “Sharma *et al*. ncomms_2015_peptides and spectra in human ventricular cardiomyocytes” and “Sharma *et al*. ncomms_2015_peptides and spectra in mouse ventricular cardiomyocytes”) were uploaded to figshare (10.6084/m9.figshare.11844972.v12)^51^. Additionally, all excel source input and analyzed output files including original uncropped images were uploaded to figshare (10.6084/m9.figshare.11844972.v12)^51^. Lastly, the original step-by-step raw mass spectrometry proteomics data for each biological replicate of mouse and human cardiomyocytes including both homogenate (labelled ‘H’) and membrane enriched (labelled TX-100 for human and NET for mouse) fractions were deposited to the ProteomeXchange Consortium with the dataset identifier (PXD017732)^52^.
