# Supplementary material for: Bioinformatic analysis of membrane and associated proteins in murine cardiomyocytes and human myocardium
Source: Sci Data. 2020 Dec 1;7:425. doi: 10.1038/s41597-020-00762-1 (PMC7708497; doi:10.1038/s41597-020-00762-1)
Supplement: Supplementary file 2 — Supplementary Information [file 41597_2020_762_MOESM2_ESM.pdf]

Bioinformatic analysis of membrane and associated proteins in murine cardiomyocytes and human myocardium

Shin-Haw Lee[1,2,†], Sina Hadipour-Lakmehsari[1,2,†], Da Hye Kim[1,2], Michelle Di Paola[1,2], Uros Kuzmanov[1,2], Saumya Shah[3,4], Joseph Jong-Hwan Lee[1,2], Thomas Kislinger[5,6], Parveen Sharma[2,7], Gavin Y. Oudit[3,4], Anthony O. Gramolini[1,2]

SUPPLEMENTARY INFORMATION

TABLE OF CONTENTS

***Supplemental Figure 1*...........2**

***Supplemental Figure 2*...........4**

***Supplemental Figure 3*...........6**

***Supplemental Figure 4*...........8**

***Supplemental Figure 5*...........10**

***Supplemental Figure 6*...........12**

***Supplemental Figure 7*...........14**

***Supplemental Figure 8*...........16**

***Supplemental Figure 9*...........18**

a

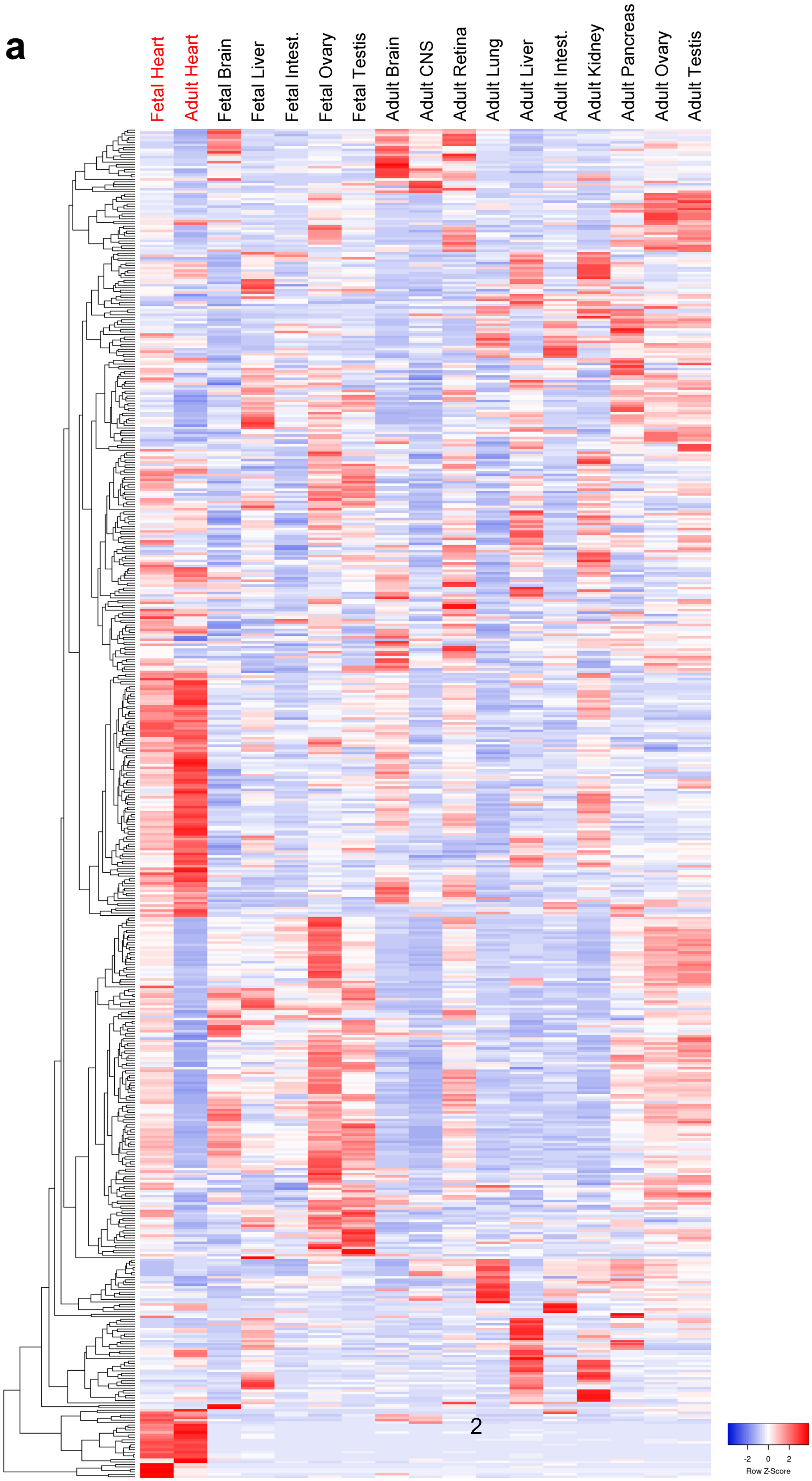

Fetal Heart
Adult Heart
Fetal Brain
Fetal Liver
Fetal Intest.
Fetal Ovary
Fetal Testis
Adult Brain
Adult CNS
Adult Retina
Adult Lung
Adult Liver
Adult Intest.
Adult Kidney
Adult Pancreas
Adult Ovary
Adult Testis
-2 0 2
Row Z-Score

Supplemental Figure 1. **Transcriptomic analysis of the 550 membrane and membrane-associated protein clusters across various tissues.** (a) Heatmap showing unsupervised clustering of mRNA transcripts of the 550 membrane-associated proteins identified across clinically defined healthy human tissues; mRNA transcript data were obtained from Human Proteome Map. All source data input and normalized output files were uploaded to figshare (https://doi.org/10.6084/m9.figshare.11844972.v12).

Cardiac-enriched (BioGPS) with previous MGI cardiac phenotype

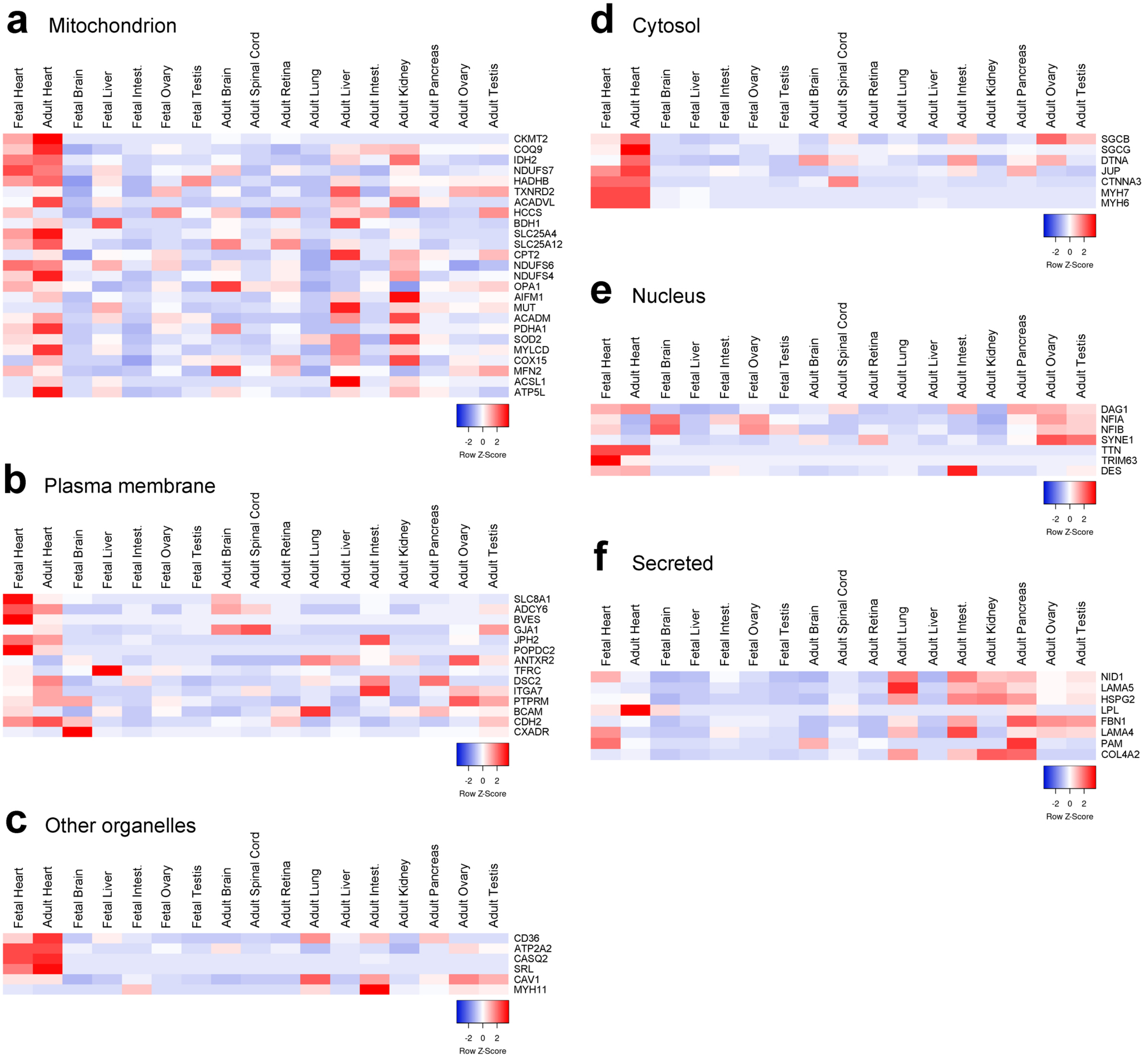

Supplemental Figure 2. **Transcriptomic analysis of cardiomyocyte-enriched membrane associated proteins with previous cardiac MGI phenotype.** Heatmaps showing mRNA transcript levels of 67 cardiomyocyte-enriched membrane proteins with previously identified cardiac MGI phenotype across clinically defined healthy human tissues; mRNA transcript data were obtained from Human Proteome Map and are presented according to their subcellular classifications in (a) mitochondrion, (b) plasma membrane, (c) other organelles (ER, golgi apparatus, peroxisomes, lysosomes), (d) cytosol, (e) nucleus, and (f) the secretory pathway. All source data input and normalized output files were uploaded to figshare (https://doi.org/10.6084/m9.figshare.11844972.v12).

Non cardiac-enriched (BioGPS) with no previous MGI cardiac phenotype

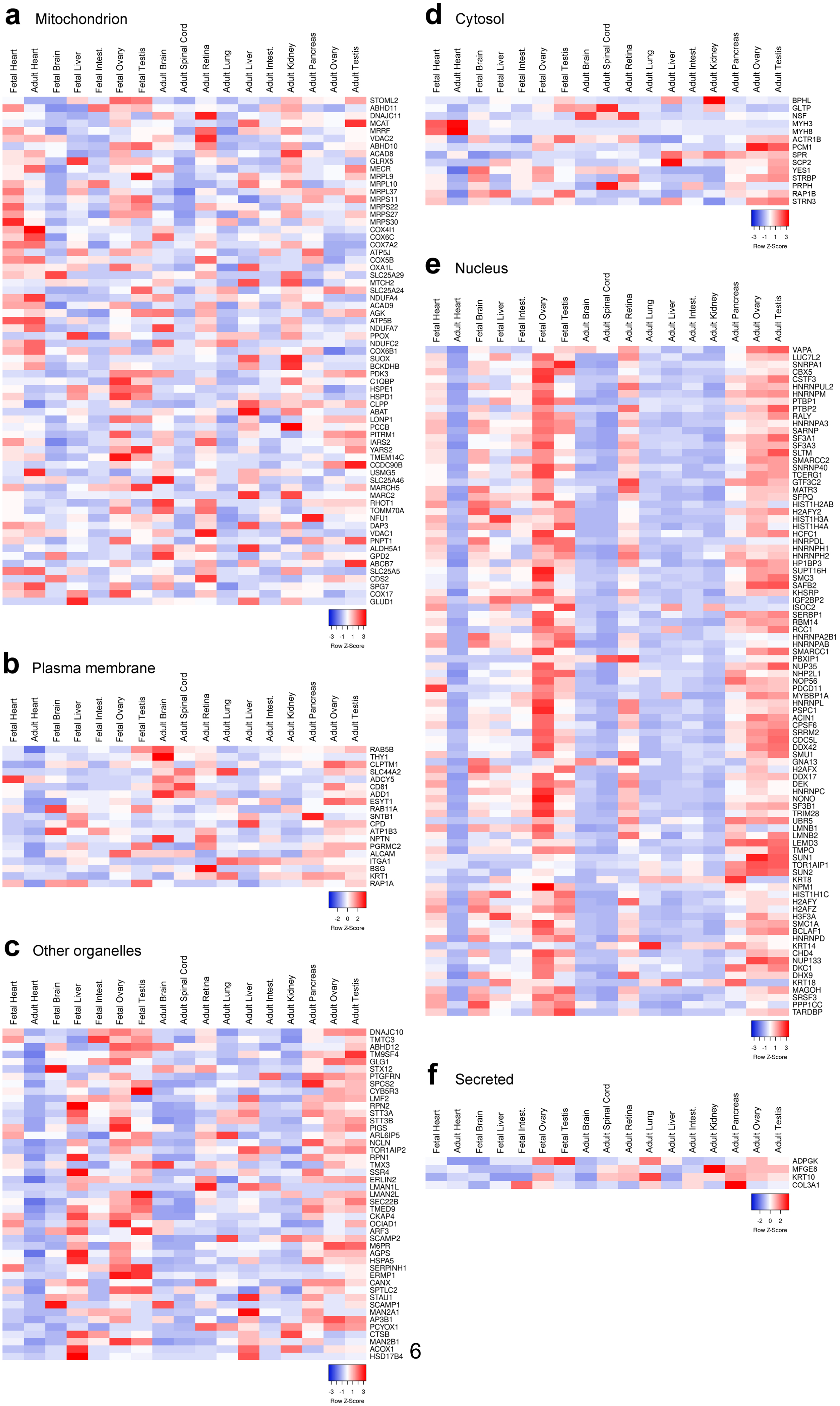

Supplemental Figure 3. **Transcriptomic analysis of non-cardiomyocyte-enriched membrane associated proteins with no previous cardiac MGI phenotype.** Heatmaps showing mRNA transcript levels of 241 non-cardiomyocyte-enriched membrane proteins with no previously identified cardiac MGI phenotype across clinically defined healthy human tissues; mRNA transcript data were obtained from Human Proteome Map and are presented according to their subcellular classifications in (a) mitochondrion, (b) plasma membrane, (c) other organelles (ER, golgi apparatus, peroxisomes, lysosomes), (d) cytosol, (e) nucleus, and (f) the secretory pathway. All source data input and normalized output files were uploaded to figshare (https://doi.org/10.6084/m9.figshare.11844972.v12).

Non cardiac-enriched (BioGPS) with previous MGI cardiac phenotype

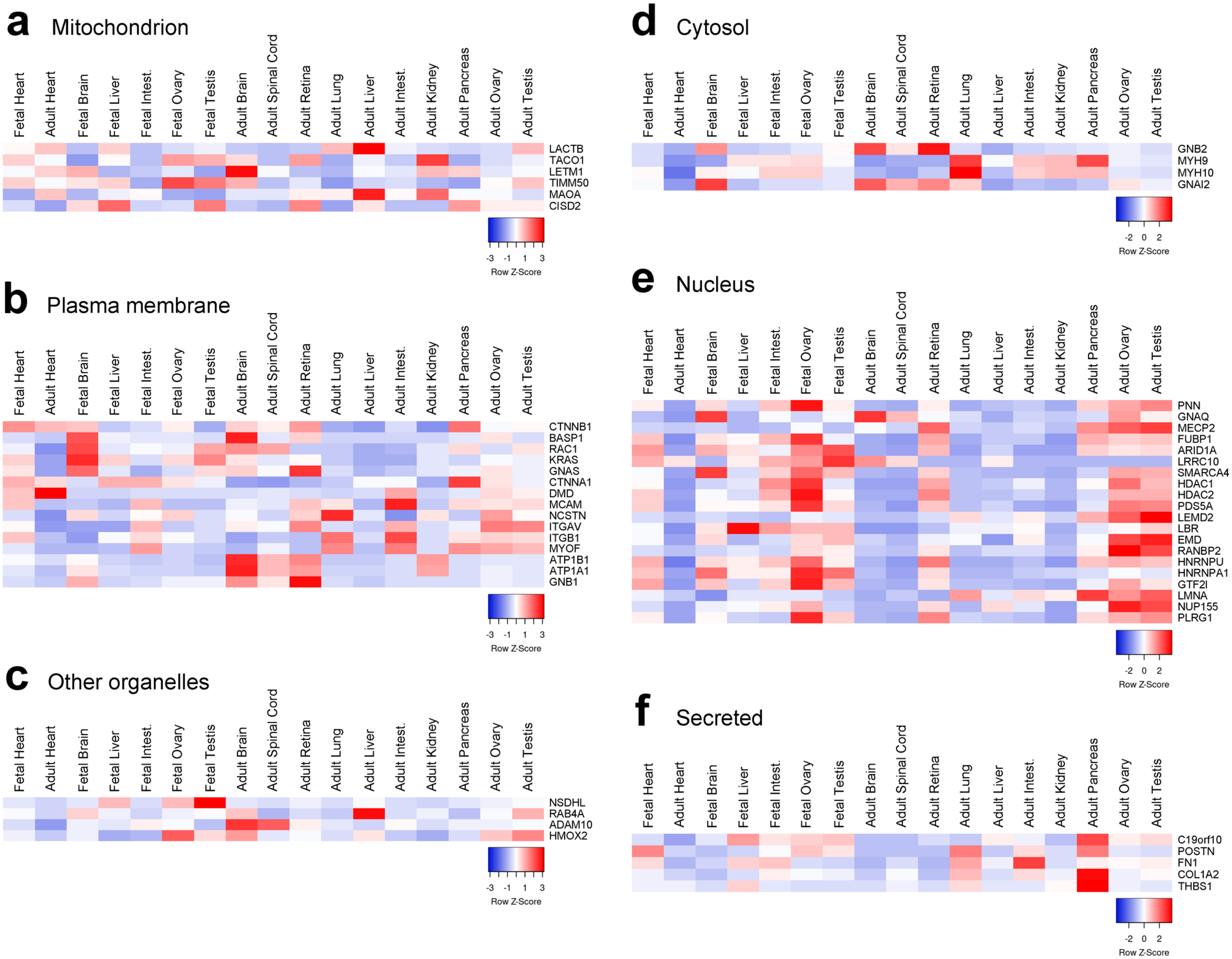

Supplemental Figure 4. **Transcriptomic analysis of non-cardiomyocyte-enriched membrane associated proteins with previous cardiac MGI phenotype.** Heatmaps showing mRNA transcript levels of 54 non-cardiomyocyte-enriched membrane proteins with previously identified cardiac MGI phenotype across clinically defined healthy human tissues; mRNA transcript data were obtained from Human Proteome Map and are presented according to their subcellular classifications in (a) mitochondrion, (b) plasma membrane, (c) other organelles (ER, golgi apparatus, peroxisomes, lysosomes), (d) cytosol, (e) nucleus, and (f) the secretory pathway. All source data input and normalized output files were uploaded to figshare (https://doi.org/10.6084/m9.figshare.11844972.v12).

a

```
                                                                        TM1
FAM162A|Human        MGSLSGLRLAAGSCFRLCERDVSSSLRLTRSSDLKRINGFCTKPQESPGAPSRT--YNRV 58
FAM162A|Rat          MWSLRGLRLAAGHCFRLCERNVSSPLRLTRNTDLKRINGFCTKPQESPKAPTQ-SYRHRV 59
FAM162A|Mouse        MWSLGGLRLAAGHCLRLYERNASSSLRFTRNTDLKRINGFCTKPQESPKTPTQ-SYRHGV 59
FAM162A|Chicken      MWG------RADKAVKLLERNIPSFLRMSRGVGPKVSRRLCSKPQEESSVQPRGRPVLRV 54
FAM162A|Cow          MGSLRGLRLVAGSCFRSCERDAFSSLRLTRNSDLKRTNGFCSKPQESPKPPDQHTYSHRV 60
FAM162A|Horse        MGSLRGLRLAAGSCFRLCERDASSSLRLTRNSDLKRINGFCTKPQESPKAPS-HTYSHRV 59
FAM162A|Monkey       MGTLRGLLLAAGSCFRLCERDVSSSLRLTRSSDLKIINGFCIKPQESPRAPSR-TYSHRV 59
FAM162A|Chimpanzee   MGSLSGLRLAAGSCFRLCERDVSSSLRLTRSSDLKRINGFCTKPQESPGAPSRT--YNRV 58
                              10        20        30        40        50        60

FAM162A|Human        PLHKPTDWQKKILIWSGRFKKEDEIPETVSLEMLDAAKNKMRVKISYLMIALTVVGCIFM 118
FAM162A|Rat          PLHKPTDFEKKILLWSGRFKKEEEIPETISFEMLDAAKNKIRVKVSYLMIALTVAGCVYM 119
FAM162A|Mouse        PLHKPTDFEKKILLWSGRFKKEEEIPETISFEMLDAAKNKLRVKVSYLMIALTVAGCIYM 119
FAM162A|Chicken      PGHKPTDWEKKCLLWAGHFKKPEDIPEVVSIDTIRAAQTTLRVKFSYVMIALTIVGCIVM 114
FAM162A|Cow          PLHKPTDWEKKILIWSGRFKKEDEIPETVSFEMLDAAKNKVRVKISYVMIALTVAGCVLM 120
FAM162A|Horse        PLHKPTDWERKILIWSGRFKKEDEIPETVSFEMLDAAKNKIRVKISYLMIALTVAGCILM 119
FAM162A|Monkey       PLHKPTDWQKKMLIWSGRFKKEDEIPETVSLEMLDAAKNKMRVKICYLMIALTVIGCIAM 119
FAM162A|Chimpanzee   PLHKPTDWQKKILIWSGRFKKEDEIPETVSLEMLDAAKNKMRVKISYLMIALTVVGCIFM 118
                              70        80        90       100       110       120
                     TM1
FAM162A|Human        VIEGKKAAQRHETLTSLN---------------LEKKARLKEEAAMKAKTE--------  154
FAM162A|Rat          VIEGKKLEPRKESPSERGGSYEGQSGLDVFVLDLEKPGIFQKPSPSGKAIERERDTVQG  178
FAM162A|Mouse        VIEGKKAAKRHESLTSLN---------------LERKARLREEAAMKAKTD--------  155
FAM162A|Chicken      VIRGKQAVKRHESLTSIN---------------LEKKAQWKEEATQSTSAKP-------  151
FAM162A|Cow          VIEGKKAARRNETLTSLN---------------LEKKARLREEAAMKAKTE--------  156
FAM162A|Horse        VIEGKKAVKRNESLTSLN---------------LEKKARLREEAAMKAKTE--------  155
FAM162A|Monkey       IIDGKKAAQRNQTLTRLN---------------LEKKARLREEAAMNAKTE--------  155
FAM162A|Chimpanzee   VIEGKKAAQRHETLTSLN---------------LERKARLKEEAAMKAKTE--------  154
                             130       140       150       160       170
```

RED indicates positions which have a single, fully conserved residue.

b

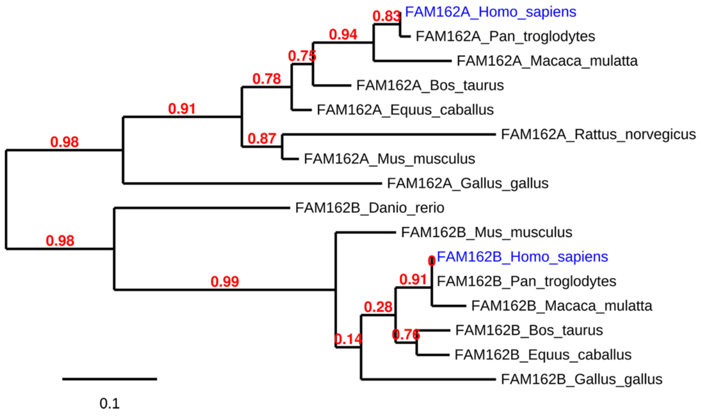

c

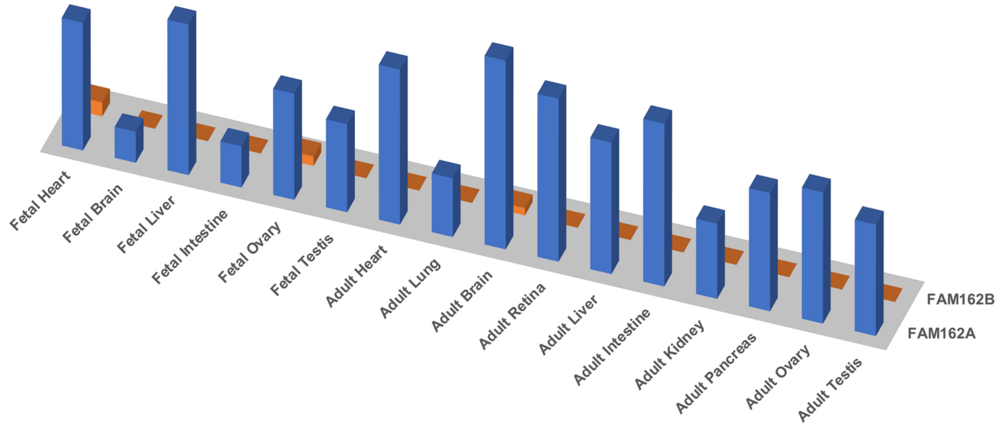

d

```
                                                           TM1              TM2
COX20|Human          MAAPPEPGEPEERKSLKLLGFLDVENTPCARHSILYGSLGSVVAGFGHFLFTSRIRRSCD   60
COX20|Rat            MAAASEPHEP-EKKPFKLLGILDVENTPCARESILYGSLGSIVTGLGHFLVTSRIRRSCD   59
COX20|Mouse          MAAAPEPHET-EKKPFKLLGILDVENTPCARESILYGSLGSIVTGLGHFLVTSRIRRSCD   59
COX20|Chicken        ---MAGEGDSEPEKSFKLLGFLDVKNVPCARESVLYGSLGSLVVGLGHFLATSRVRRSCD   57
COX20|Cow            -------MAA-APEPFKLLGILDVENIPCARDSVLYGSLGSVVAGLGHFLLTSRIRRSCD   52
COX20|Monkey         MAAAPEPGEPKERKAFKLLGFLDVENIPCARDSILYGSLGSIVAGFGHFLFTSRIKRSCD   60
COX20|Chimpanzee     MAAAPEPGEPEERKSVKLLGFLDVENTPCARHSILYGSLGSAVAGFGHFLFTSRIRRSCD   60
COX20|Frog           --MAGQEGEVVKEKSFKLLGIIDVQNTPCARESILYGTVGSLVLGLGHFLATSRVRRSCD   58
COX20|Zebrafish      -----MTEEDGKTQGMKVLGILDIHNTPCAREAILHGAAGSVAAGLLHFLATSRVKRSFD   55
                              10        20        30        40        50        60
                       TM2
COX20|Human          VGVGGFILVTLGCWFHCRYNYAKQRIQERIAREEIKKKILYEGTHLDPERKHNGSSSN--   118
COX20|Rat            VGVGGFILVTLGCWFHCRYNFAKQRIQERIAREGIKNKILYESTHLDPERKTKSSNSS--   117
COX20|Mouse          VGVGGFILVTLGCWFHCRYNFAKQRIQERIAREGIKNKILYESTHLDPERKMKTNNSS--   117
COX20|Chicken        FAVGGFICTMLGYWFYCRYNLAQQRIRQRMLKEGMRNKMLFEGSSFDPEKKQTGNERSNS   117
COX20|Cow            VGVGGFIVVTLGCWFHCRYNYAKLRIQERLAREGIKNKILYESTHLDPARKQTNGGSSSS   112
COX20|Monkey         VGVGGFILVTLGCWFHCRYNYAKQRIRERIAREEIKKKILYEGTHLDPERKHNSNSSN--   118
COX20|Chimpanzee     VGVGGFILVTLGCWFHCRYNYAKQRIQERIAREEIKKKILYEGTHLDPERKHNGSSSN--   118
COX20|Frog           VAVGGYILTTLGCWMHCRYNNAKVRIQQKMLQEGIKNRILFEGSSIDPNTRKNTTDSKT-   117
COX20|Zebrafish      VGVAGFMITTLGSWFYCRYNNAKLRFQQRIIQEGLKNKVFYEGTDLDPTLKKTGDK----   111
                              70        80        90       100       110       120
```

RED indicates positions which have a single, fully conserved residue.

Supplemental Figure 5. **FAM162A and COX20 share high degrees of homology throughout evolution.** (a) A multi-species alignment of FAM162A from selected vertebrates shows 65% peptide conservation throughout evolution. (b) Phylogenetic analysis of the FAM162 family of proteins displays clustering of mammalian taxa with conservation throughout multiple species. (c) mRNA transcript levels of FAM162A and FAM162B obtained from Human Proteome Map across various healthy human organ tissues in transcriptomic analyses. (d) A multi-species alignment of COX20 from vertebrates demonstrates 69% peptide conservation throughout evolution. TM indicates the transmembrane domains identified. All source data input and normalized output files were uploaded to figshare (https://doi.org/10.6084/m9.figshare.11844972.v12).

a

```
                                                 TM1
MCT1/SLC16A|Human        MPPAVGGPVGYTPPDGGWGWAVVIGAFISIGFSYAFPKSITVFFKEIEGIFHATTSEVSW    60
MCT1/SLC16A|Rat          MPPAIGGPVGYTPPDGGWGWAVVVGAFISIGFSYAFPKSITVFFKEIEIIFSATTSEVSW    60
MCT1/SLC16A|Mouse        MPPAIGGPVGYTPPDGGWGWAVLVGAFISIGFSYAFPKSITVFFKEIEVIFSATTSEVSW    60
MCT1/SLC16A|Chicken      MPPAIGGPVGYTPPEGGWGWAVVVGAFISIGFSYAFPKSITVFFKEIEVIFNASSSKVSW    60
MCT1/SLC16A|Cow          MPPAVGGPVGYTPPDGGWGWAVVIGAFISIGFSYAFPKSITVFFKEIEGIFNATTSEVSW    60
MCT1/SLC16A|Horse        MPPAVGGPVGYTPPDGGWGWAVVVGAFISIGFSYAFPKSITVFFKEIESIFNATTSEVSW    60
MCT1/SLC16A|Monkey       MPPAVGGPVGYTPPDGGWGWAVVIGAFISIGFSYAFPKSITVFFKEIESIFHATTSEVSW    60
MCT1/SLC16A|Chimpanzee   MPPAVGGPVGYTPPDGGWGWAVVIGAFISIGFSYAFPKSITVFFKEIEGIFHATTSEVSW    60
MCT1/SLC16A|Frog         MAPAVGGPVGYTPPEGGWGWVVVVAAFVSIGFSYAFPKSITVFFKDIEAIFGATSSEVSW    60
MCT1/SLC16A|Zebrafish    MPPATGGPVGYTPPEGGWGWAVVVGAFISIGFSYAFPKSITVFFKEIEVIFNATSSQVSW    60
                                  10        20        30        40        50        60
                                                      TM2                    TM3
MCT1/SLC16A|Human        ISSIMLAVMYGGGPISSILVNKYGSRIVMIVGGCLSGCGLIAASFCNTVQQLYVCIGVIG   120
MCT1/SLC16A|Rat          ISSIMLAVMYAGGPISSILVNKYGSRPVMIAGGCLSGCGLIAASFCNTVQELYFCIGVIG   120
MCT1/SLC16A|Mouse        ISSIMLAVMYAGGPISSILVNKYGSRPVMIAGGCLSGCGLIAASFCNTVQELYLCIGVIG   120
MCT1/SLC16A|Chicken      ISSIMLAVMYAGGPISSILVNKYGSRPIMIVGGCLSGCGLIAASFCNTVEELYFCVGVVG   120
MCT1/SLC16A|Cow          ISSIMLAVMYGGGPISSVLVNKYGSRPVMIVGGILSGSGLIAASFCNTVQELYFSVGVIG   120
MCT1/SLC16A|Horse        ISSIMLAVMYGGGPISSILVNKYGSRPIMILGGCLSGSGLIAASFCNTVQELYLCIGVIG   120
MCT1/SLC16A|Monkey       ISSIMLAVMYGGGPISSILVNKYGSRIVMIIGGCLSGCGLIAASFCNTVQELYFCIGFVG   120
MCT1/SLC16A|Chimpanzee   ISSIMLAVMYGGGPISSILVNKYGSRIVMIIGGCLSGCGLIAASFCNTVQQLYFCIGVIG   120
MCT1/SLC16A|Frog         ISSIMLAVMYAGGPISSVLVNKYGSRPVMIAGGCLAGTGLIAASFCNTVAELYLCIGVVG   120
MCT1/SLC16A|Zebrafish    ISSIMLAVMYAGGPISSILVNKFGSRPIMIAGGCLSGTGLVAASFCDTVEGLYFCVGVIG   120
                                  70        80        90       100       110       120
                            TM3                 TM4                     TM5
MCT1/SLC16A|Human        GLGLAFNLNPALTMIGKYFYKRRPLANGLAMAGSPVFLCTLAPLNQVFFGIFGWRGSFLI   180
MCT1/SLC16A|Rat          GLGLAFNLNPALTMIGKYFYKKRPLANGLAMAGSPVFLSTLAPLNQAFFGIFGWRGSFLI   180
MCT1/SLC16A|Mouse        GLGLAFNLNPALTMIGKYFYKKRPLANGLAMAGSPVFLSTLAPLNQAFFDIFDWRGSFLI   180
MCT1/SLC16A|Chicken      GLGLAFNLNPALTMIGKYFFKKRPLANGLAMAGSPVFLSTLAPVNQLFFGVFGWRGSFLI   180
MCT1/SLC16A|Cow          GLGLAFNLNPALTMIGKYFYKRRPLANGLAMAGSPVFLSTLAPLNQAFFMIYGWRGSFLI   180
MCT1/SLC16A|Horse        GLGLAFNLNPALTMIGKYFYKRRPLANGLAMAGSPVFLSTLAPLNQAFFGIFGWRGSFLI   180
MCT1/SLC16A|Monkey       GLGLAFNLNPALTMIGKYFYKRRPLANGLAMAGSPVFLCTLAPLNQVFFGIFGWRGSFLI   180
MCT1/SLC16A|Chimpanzee   GLGLAFNLNPALTMIGKYFYKRRPLANGLAMAGSPVFLCTLAPLNQVFFGIFGWRGSFLI   180
MCT1/SLC16A|Frog         GFGLAFNLNPALTMIGKYFFKKRPIANGLAMAGSPVFLSTLAPLNQYFYSIFGWRGSFLI   180
MCT1/SLC16A|Zebrafish    GLGLAFNLNPALTMIGKYFYKRRPIANGIAMAGSPVFLSTLAPLNSWLFDQFGWRGSFLI   180
                                 130       140       150       160       170       180
                              TM5
MCT1/SLC16A|Human        LGGLLLNCCVAGALMRPIGPKPTKAGKDKSKASLEKAGKSGVKKDLHDANTDLIGRHPKQ   240
MCT1/SLC16A|Rat          LGGLLLNCCVAGSLMRPIGPQQGKVEKLKSKESLQEAGK-------SDANTDLIGGSPKG   233
MCT1/SLC16A|Mouse        LGGLLLNCCVAGSLMRPIGPEQVKLEKLKSKESLQEAGK-------SDANTDLIGGSPKG   233
MCT1/SLC16A|Chicken      LGGLLLNCCVAGSLMRPIGPKPDQLKKEPTKEVLQEAGKA-VKKGDGDTSTDLIGGKTKK   239
MCT1/SLC16A|Cow          LGGLLLNCCVAGALMRPIGPKPTTAEKEKSKGSLQEAGKYETKKGASDANTDLIGGNPKE   240
MCT1/SLC16A|Horse        LGGFLLNCCVAGALMRPIGPKPTNAKKERSKESLQEAGKPDAQKGAGDANTDLIGGYPKE   240
MCT1/SLC16A|Monkey       LGGLLLNCCVAGALMRPIGPKPTKAGKDKSKASLQKAGKSGVKKGRHDANTDLIGRHPKR   240
MCT1/SLC16A|Chimpanzee   LGGLLLNCCVAGALMRPIGPKPTKAGKNKSKASLEKAGKSGVKKDLHDANTDLIGRHPKQ   240
MCT1/SLC16A|Frog         LGGLLFNCCVAGSLMRPIGPKPEDTKKKVTKEVLEEAGKCV-SKEDGDAHKDLIEGKAK-   238
MCT1/SLC16A|Zebrafish    LGGALLNCCVAGSLMRPIGPKPQPAIPKEDA-----------------------EIKPQ   216
                                 190       200       210       220       230       240
                                                    TM6                    TM7
MCT1/SLC16A|Human        EKRSVFQTINQFLDLTLFTHRGFLLYLSGNVIMFFGLFAPLVFLSSYGKSQHYSSEKSAF   300
MCT1/SLC16A|Rat          EKLSVFQTVNKFLDLSLFTHRGFLLYLSGNVVMFFGLFTPLVFLSNYGKSKHFSSEKSAF   293
MCT1/SLC16A|Mouse        EKLSVFQTINKFLDLSLFTHRGFLLYLSGNVVMFFGLFTPLVFLSSYGKSKDFSSEKSAF   293
MCT1/SLC16A|Chicken      EKSTLFQTINKFLDLSLFTHRGFLLYLSGNVIMFFGLFTPLVFLSNYAKSKKIANESAAF   299
MCT1/SLC16A|Cow          EKKSIFQTLNTFLDLSLFKHRGFLLYLSGNVLMFFGLFTPLVFLSNYGKSKHYSSEKAAF   300
MCT1/SLC16A|Horse        EKQSVFQTINKFLDLSLFTHRGFLLYLSGNVLMFFGLFTPLVFLSNYGKSQHYSSEKSAF   300
MCT1/SLC16A|Monkey       EKRSVFQTINQFLDLTLFTHRGFLLYLSGNVIMFFGLFAPLVFLSSYGKSQHYSSEKSAF   300
MCT1/SLC16A|Chimpanzee   EKRSVFQTINQFLDLTLFTHRGFLLYLSGNVIMFFGLFAPLVFLSSYGKSQHYSSEKSAF   300
MCT1/SLC16A|Frog         TKPTLFQTINKFLDLSLFTHRGFLLYLSGHMIMFFGLFAPLVFLSNYAKSKNISPESAAF   298
MCT1/SLC16A|Zebrafish    EKKTVMQTINGFLDLTLFTHRGFLLYLLGNVIMFFGLFAPLVFLSNYAKSKHISKDKAAF   276
                                 250       260       270       280       290       300
                                TM7                    TM8                  TM9
MCT1/SLC16A|Human        LLSILAFVDMVARPSMGLVANTKPIRPRIQYFFAASVVANGVCHMLAPLSTTYVGFCVYA   360
MCT1/SLC16A|Rat          LLSILAFVDMVARPSMGLAANTRWIRPRVQYFFAASVVANGVCHLLAPLSTTYVGFCIYA   353
MCT1/SLC16A|Mouse        LLSILAFVDMVARPSMGLAANTKWIRPRIQYFFAASVVANGVCHLLAPLSTTYVGFCVYA   353
MCT1/SLC16A|Chicken      LLSILAFVDMVARPSMGLVANTKWIRPRVQYFFAISVIYNGVCHLLAPMSTTYAGFCIYA   359
MCT1/SLC16A|Cow          LLSILAFVDMVARPSMGLVANTKWVRPRVQYFFAASIIANGLCHLAAPLSSTYIELCIYA   360
MCT1/SLC16A|Horse        LLSILAFVDMVARPSMGLVANTKWIRPRVQYFFAASIVANGVCHLLAPLSSSYIGFCVYA   360
MCT1/SLC16A|Monkey       LLSILAFVDMVARPSMGLVANTKPIRPRIQYFFAASIVANGVCHMLAPLSTTYVGFCVYA   360
MCT1/SLC16A|Chimpanzee   LLSILAFVDMVARPSMGLVANTKPIRPRIQYFFAASVVANGVCHMLAPLSTTYVGFCVYA   360
MCT1/SLC16A|Frog         LLSILAIVDMIARPSMGIVANTKWVRPKIQYFFAVAVLYNGICHLLVPLSTSYVGFCIYA   358
MCT1/SLC16A|Zebrafish    LLSILAFTDMIARPSMGLVANTRWVRPRIQYFFAASVLYNGVCHLLAPLSTDYLGFAIYA   336
                                 310       320       330       340       350       360
                              TM9                      TM10
MCT1/SLC16A|Human        GFFGFAFGWLSSVLFETLMDLVGPQRFSSAVGLVTIVECCPVLLGPPLLGRLNDMYGDYK   420
MCT1/SLC16A|Rat          GVFGFAFGWLSSVLFETLMDLVGPQRFSSAVGLVTIVECCPVLLGPPLLGRLNDMYGDYK   413
MCT1/SLC16A|Mouse        GVFGFAFGWLSSVLFETLMDLIGPQRFSSAVGLVTIVECCPVLLGPPLLGRLNDMYGDYK   413
MCT1/SLC16A|Chicken      GFFGFAFGWLSSVLFETLMDLVGAQRFSSAVGLVTIVECCPVLLGPPLLGKLNDMYGDYK   419
MCT1/SLC16A|Cow          GFFGFAFGWLSSVLFETLMDLVGPQRFSSAVGLVTIVECCPVLLGPPVLGRLNDIYGDYK   420
MCT1/SLC16A|Horse        GFFGFAFGWLSSVLFETLMDLVGPQRFSSAVGLVTIVECCPVLLGPPLLGRLNDIYGDYK   420
MCT1/SLC16A|Monkey       GFFGFAFGWLSSVLFETLMDLVGPQRFSSAVGLVTIVECCPVLLGPPLLGRLSDMYGDYK   420
MCT1/SLC16A|Chimpanzee   GFFGFAFGWLSSVLFETLMDLVGPQRFSSAVGLVTIVECCPVLLGPPLLGRLNDMYGDYK   420
MCT1/SLC16A|Frog         GFFGFAFGWLSSVLFETLMDLVGAQRFSSAVGLVTIVECCPVLLGPPLLGRLNDIYGDYK   418
MCT1/SLC16A|Zebrafish    VFFGVAFGWLSSVLFETLMDLVGAQRFSSAVGLVTIVECAPVLLGPPLLGRLNDIYDDYK   396
                                 370       380       390       400       410       420
                                TM11
MCT1/SLC16A|Human        YTYWACGVVLIISGIYLFIGMGINYRLLAKEQKANE--QKKESKEEETSIDVAGKPNEVT   478
MCT1/SLC16A|Rat          YTYWACGVILIIAGLYLFIGMGINYRLVAKEQKAEE-K-KRDGKEDETSTDVDEKPKKTM   471
MCT1/SLC16A|Mouse        YTYWACGVILIIAGIYLFIGMGINYRLLAKEQKAEE-KQKREGKEDEASTDVDEKPKETM   472
MCT1/SLC16A|Chicken      YTYWACGIILIVSGIYLFIGMGINYRLVAKEQKAAEEKTKNEGKEEETNIDEAEKQKEAN   479
MCT1/SLC16A|Cow          YTYWACGIILIVAGIYLFIGMGINYRLLEKEQKAEK-QQKKESKDEETNVDVAEKPKEVI   479
MCT1/SLC16A|Horse        YTYWACGIILIVAGIYLFIGMGINYHLLAKEQKAEK-KQRKESKEVETGVDVAEKPKEVT   479
MCT1/SLC16A|Monkey       YTYWACGVVLIISGIYLFIGMGINYRLLAKEQKANE--QKKESKEEETSIDVAGKPKEVT   478
MCT1/SLC16A|Chimpanzee   YTYWACGVVLIISGIYLFIGMGINYRLLAKEQKANE--QKMESKEEETSIDVAGKPNEVT   478
MCT1/SLC16A|Frog         YTYQACGIVLIVASVYLFIGMTIHYRLLAKEQKAEQKKAQEANAGASENK--------VN   470
MCT1/SLC16A|Zebrafish    YTYQGCGIVLVIASIFLFVGMGINYRLVDKEKKEEEKRAKLEDKDEETNLDNALNEKEKE   456
                                 430       440       450       460       470       480

MCT1/SLC16A|Human        KAAESPDQ-KDTDGGPKEEESPV    500
MCT1/SLC16A|Rat          KETQSPAPLQNSSGDPAEEESPV    494
MCT1/SLC16A|Mouse        KAAQSP--QQHSSGDPTEEESPV    493
MCT1/SLC16A|Chicken      NDVATLPQ-KSTEDGVKEEESHM    501
MCT1/SLC16A|Cow          DAAESPEH-KATEEDPKEAESPV    501
MCT1/SLC16A|Horse        NAAGSPEQ-KGTEGDPKEESPL-    500
MCT1/SLC16A|Monkey       KAAESPDQ-KDTEEGPKEEDSPV    500
MCT1/SLC16A|Chimpanzee   KAAESPDQ-KDTEGGPKEEESPV    500
MCT1/SLC16A|Frog         ELSVLP---QNSDDVLKEDESHI    490
MCT1/SLC16A|Zebrafish    ADGVV------------------    461
                                 490       500
```

RED indicates positions which have a single, fully conserved residue.

Supplemental Figure 6. (a) A multi-species alignment of MCT1 from selected vertebrates demonstrates 78% peptide conservation throughout evolution. TM indicates the transmembrane domains identified.

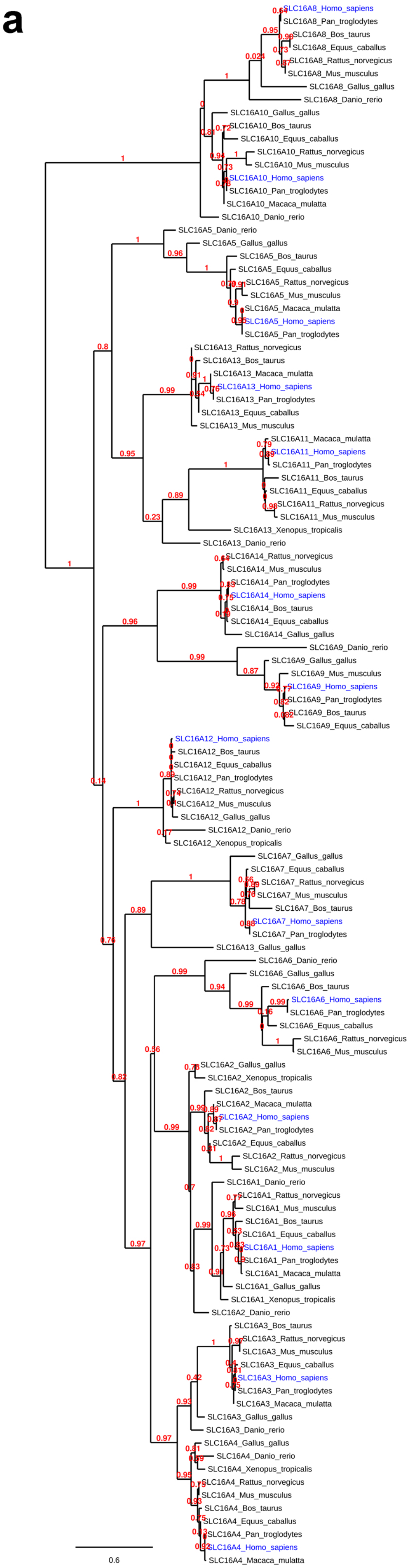
a
SLC16A8_Homo_sapiens
SLC16A8_Pan_troglodytes
SLC16A8_Bos_taurus
SLC16A8_Equus_caballus
SLC16A8_Rattus_norvegicus
SLC16A8_Mus_musculus
SLC16A8_Gallus_gallus
SLC16A8_Danio_rerio
SLC16A10_Gallus_gallus
SLC16A10_Bos_taurus
SLC16A10_Equus_caballus
SLC16A10_Rattus_norvegicus
SLC16A10_Mus_musculus
SLC16A10_Homo_sapiens
SLC16A10_Pan_troglodytes
SLC16A10_Macaca_mulatta
SLC16A10_Danio_rerio
SLC16A5_Danio_rerio
SLC16A5_Gallus_gallus
SLC16A5_Bos_taurus
SLC16A5_Equus_caballus
SLC16A5_Rattus_norvegicus
SLC16A5_Mus_musculus
SLC16A5_Macaca_mulatta
SLC16A5_Homo_sapiens
SLC16A5_Pan_troglodytes
SLC16A13_Rattus_norvegicus
SLC16A13_Bos_taurus
SLC16A13_Macaca_mulatta
SLC16A13_Homo_sapiens
SLC16A13_Pan_troglodytes
SLC16A13_Equus_caballus
SLC16A13_Mus_musculus
SLC16A11_Macaca_mulatta
SLC16A11_Homo_sapiens
SLC16A11_Pan_troglodytes
SLC16A11_Bos_taurus
SLC16A11_Equus_caballus
SLC16A11_Rattus_norvegicus
SLC16A11_Mus_musculus
SLC16A13_Xenopus_tropicalis
SLC16A13_Danio_rerio
SLC16A14_Rattus_norvegicus
SLC16A14_Mus_musculus
SLC16A14_Pan_troglodytes
SLC16A14_Homo_sapiens
SLC16A14_Bos_taurus
SLC16A14_Equus_caballus
SLC16A14_Gallus_gallus
SLC16A9_Danio_rerio
SLC16A9_Gallus_gallus
SLC16A9_Mus_musculus
SLC16A9_Homo_sapiens
SLC16A9_Pan_troglodytes
SLC16A9_Bos_taurus
SLC16A9_Equus_caballus
SLC16A12_Homo_sapiens
SLC16A12_Bos_taurus
SLC16A12_Equus_caballus
SLC16A12_Pan_troglodytes
SLC16A12_Rattus_norvegicus
SLC16A12_Mus_musculus
SLC16A12_Gallus_gallus
SLC16A12_Danio_rerio
SLC16A12_Xenopus_tropicalis
SLC16A7_Gallus_gallus
SLC16A7_Equus_caballus
SLC16A7_Rattus_norvegicus
SLC16A7_Mus_musculus
SLC16A7_Bos_taurus
SLC16A7_Homo_sapiens
SLC16A7_Pan_troglodytes
SLC16A13_Gallus_gallus
SLC16A6_Danio_rerio
SLC16A6_Gallus_gallus
SLC16A6_Bos_taurus
SLC16A6_Homo_sapiens
SLC16A6_Pan_troglodytes
SLC16A6_Equus_caballus
SLC16A6_Rattus_norvegicus
SLC16A6_Mus_musculus
SLC16A2_Gallus_gallus
SLC16A2_Xenopus_tropicalis
SLC16A2_Bos_taurus
SLC16A2_Macaca_mulatta
SLC16A2_Homo_sapiens
SLC16A2_Pan_troglodytes
SLC16A2_Equus_caballus
SLC16A2_Rattus_norvegicus
SLC16A2_Mus_musculus
SLC16A1_Danio_rerio
SLC16A1_Rattus_norvegicus
SLC16A1_Mus_musculus
SLC16A1_Bos_taurus
SLC16A1_Equus_caballus
SLC16A1_Homo_sapiens
SLC16A1_Pan_troglodytes
SLC16A1_Macaca_mulatta
SLC16A1_Gallus_gallus
SLC16A1_Xenopus_tropicalis
SLC16A2_Danio_rerio
SLC16A3_Bos_taurus
SLC16A3_Rattus_norvegicus
SLC16A3_Mus_musculus
SLC16A3_Equus_caballus
SLC16A3_Homo_sapiens
SLC16A3_Pan_troglodytes
SLC16A3_Macaca_mulatta
SLC16A3_Gallus_gallus
SLC16A3_Danio_rerio
SLC16A4_Gallus_gallus
SLC16A4_Danio_rerio
SLC16A4_Xenopus_tropicalis
SLC16A4_Rattus_norvegicus
SLC16A4_Mus_musculus
SLC16A4_Bos_taurus
SLC16A4_Equus_caballus
SLC16A4_Pan_troglodytes
SLC16A4_Homo_sapiens
SLC16A4_Macaca_mulatta
0.6

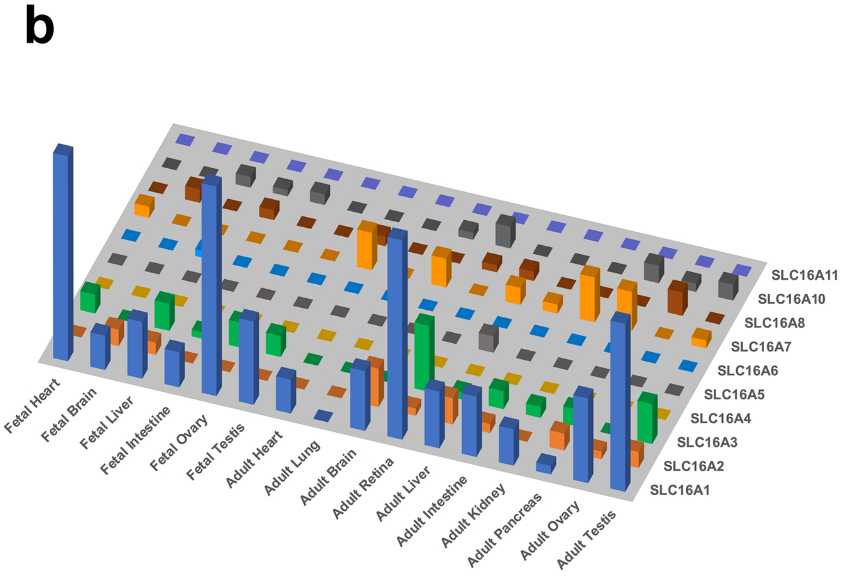
b
SLC16A11
SLC16A10
SLC16A8
SLC16A7
SLC16A6
SLC16A5
SLC16A4
SLC16A3
SLC16A2
SLC16A1
Fetal Heart
Fetal Brain
Fetal Liver
Fetal Intestine
Fetal Ovary
Fetal Testis
Adult Heart
Adult Lung
Adult Brain
Adult Retina
Adult Liver
Adult Intestine
Adult Kidney
Adult Pancreas
Adult Ovary
Adult Testis

Supplemental Figure 7. **Transcriptomic and phylogenetic analyses of the SLC16A/MCT family of proteins.** (a) Phylogenetic analysis of the SLC16A/MCT family of proteins identifies phylogenetically distinct groups and shows evolutionary conservation within clustered mammalian taxa. (b) mRNA transcript levels of SLC16A/MCT family of proteins obtained from Human Proteome Map demonstrates dominant expression of SLC16A1/MCT1 across various healthy human organ tissues in transcriptomic analyses. All source data input and normalized output files were uploaded to figshare (https://doi.org/10.6084/m9.figshare.11844972.v12).

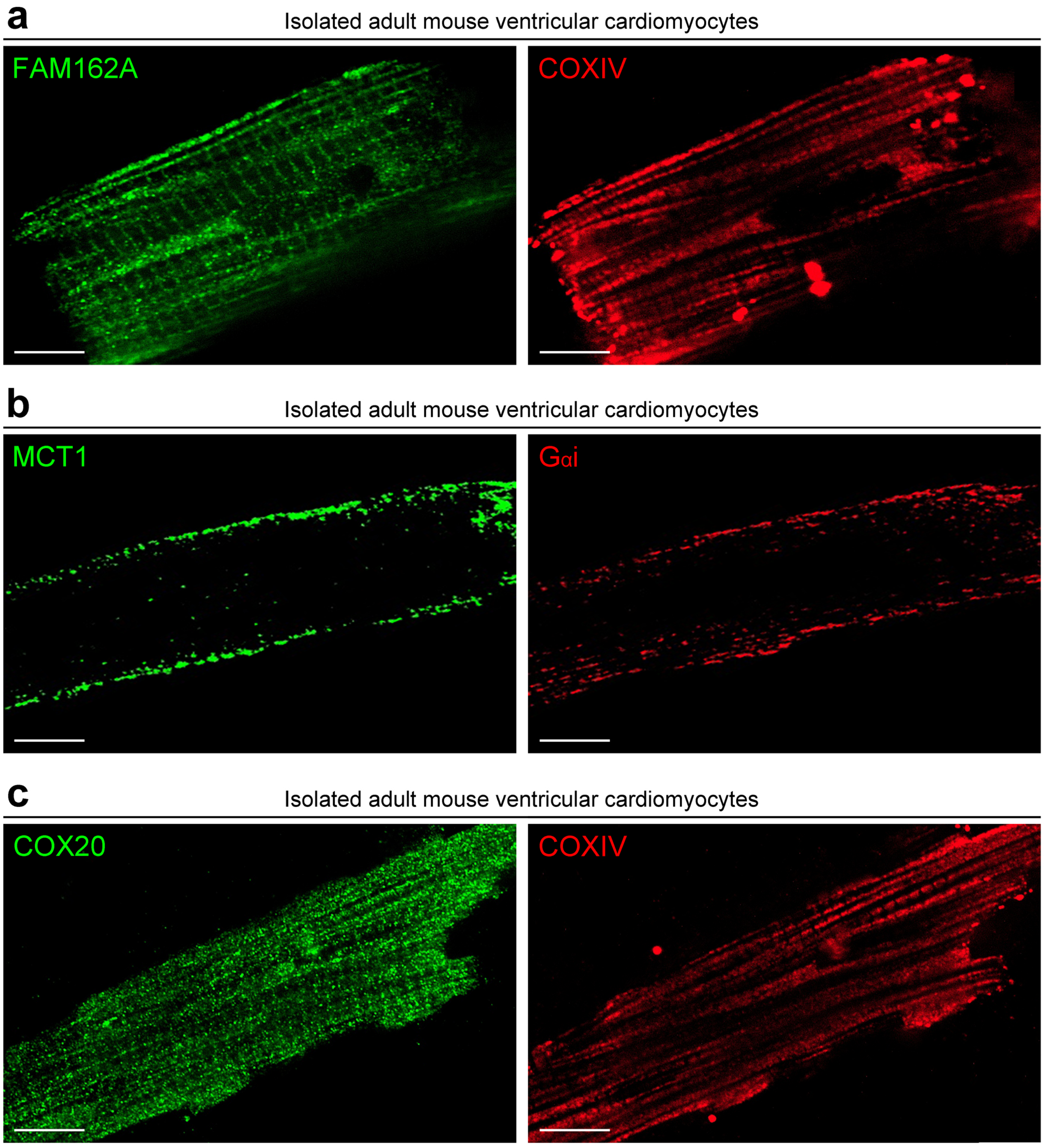
a
Isolated adult mouse ventricular cardiomyocytes
FAM162A
COXIV
b
Isolated adult mouse ventricular cardiomyocytes
MCT1
Gαi
c
Isolated adult mouse ventricular cardiomyocytes
COX20
COXIV

Supplemental Figure 8. **Co-immunofluorescence analysis of FAM162A, MCT1, and COX20 with known mitochondrial marker, COXIV and known plasma membrane protein, Gαi in isolated adult mouse cardiomyocytes.** (a) Immunofluorescence analysis of FAM162A (green) co-stained with mitochondrial protein, COXIV (red) in acutely isolated adult mouse cardiomyocytes. Scale, 10 μm. (b) Immunofluorescence analysis of MCT1 (green) co-stained with known plasma membrane protein, Gαi (red) in acutely isolated adult mouse cardiomyocytes. Scale, 10μm. (c) Immunofluorescence analysis of COX20 (green) co-stained with mitochondrial protein, COXIV (red) in acutely isolated adult mouse cardiomyocytes. Scale, 10μm. All images shown are representative of approximately 30-40 total images captured per condition, n=3 independent biological replicates. All original uncropped microscopy images were uploaded to figshare (https://doi.org/10.6084/m9.figshare.11844972.v12).

Fig. 5a

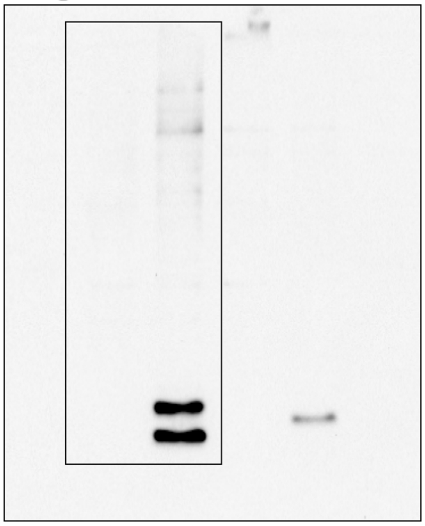

IB:DDK

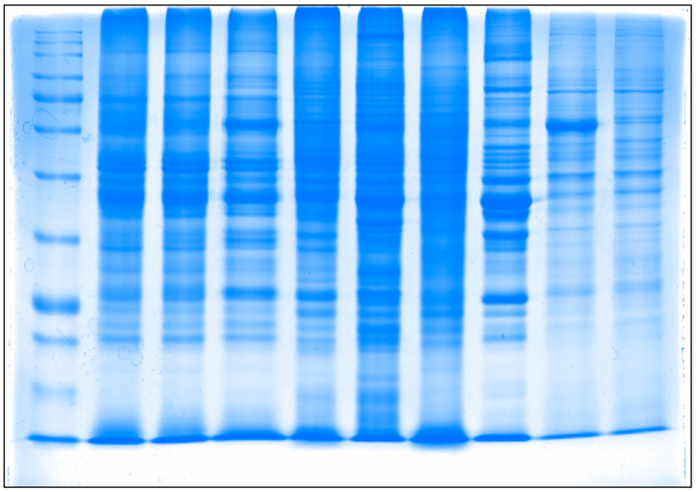

Coomassie Blue

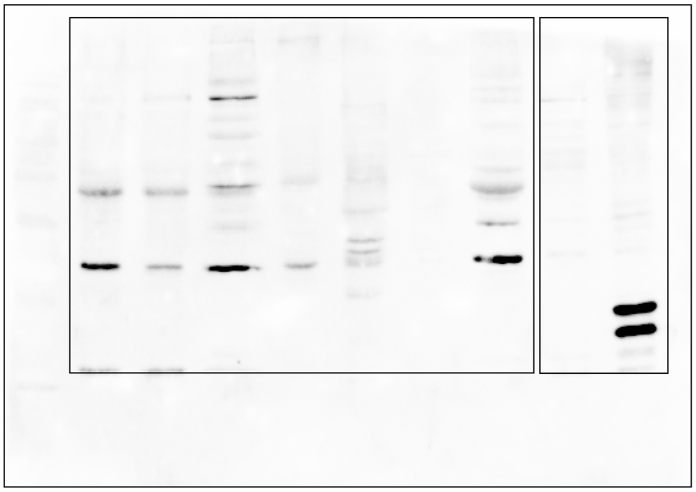

IB:FAM162A

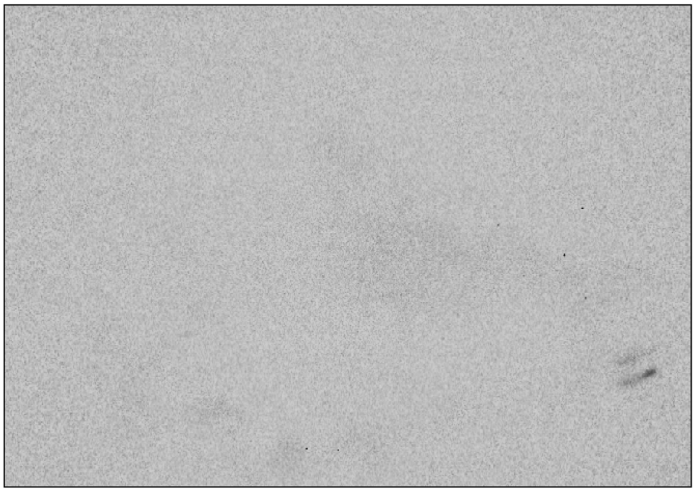

IB:FAM162 Immunodepletion

Fig. 5b

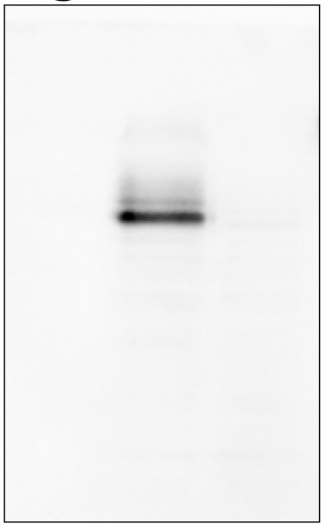

IB:DDK

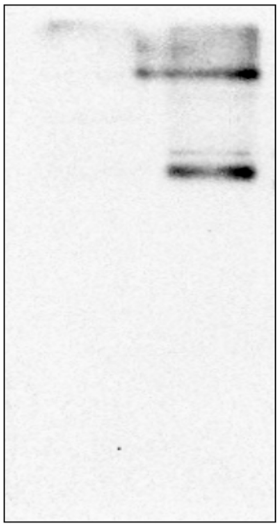

IB:MCT1

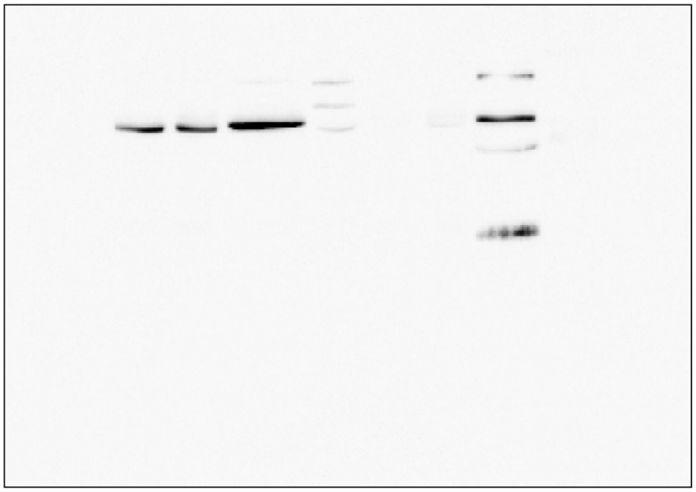

IB:MCT1

Fig. 5b cont.

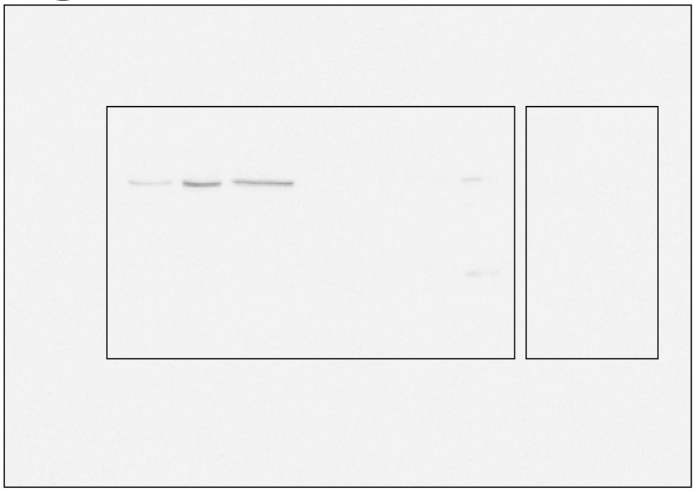

IB:MCT1 Immunodepletion

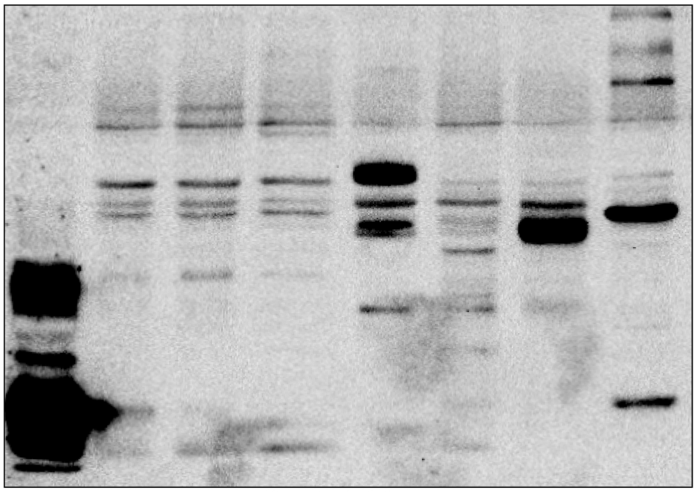

IB:COX20

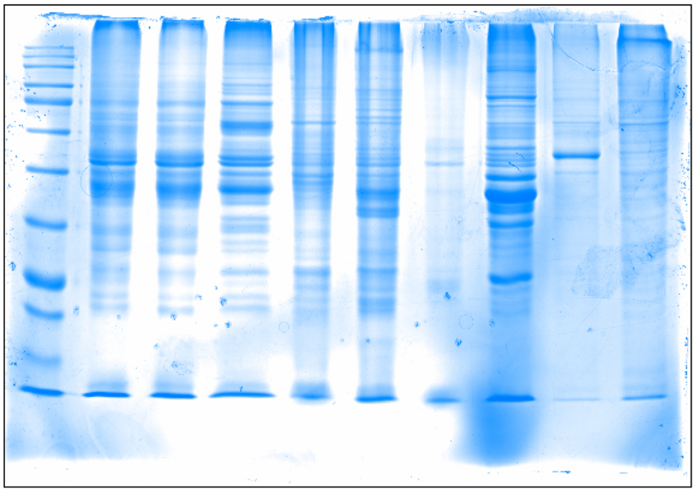

Coomassie Blue

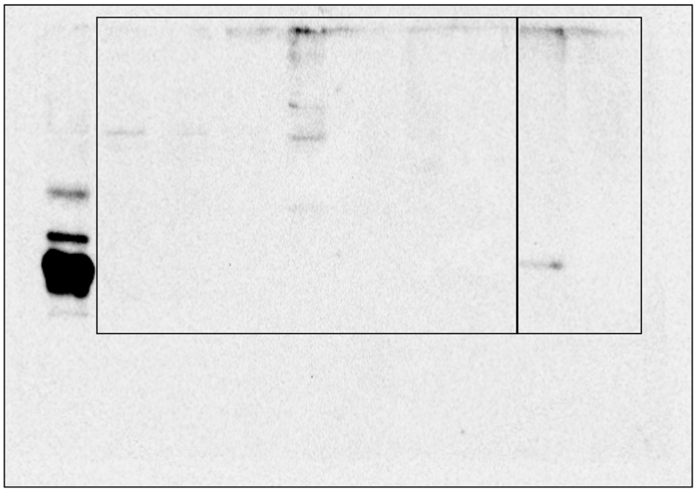

IB:COX20 Immunodepletion

Fig. 5c

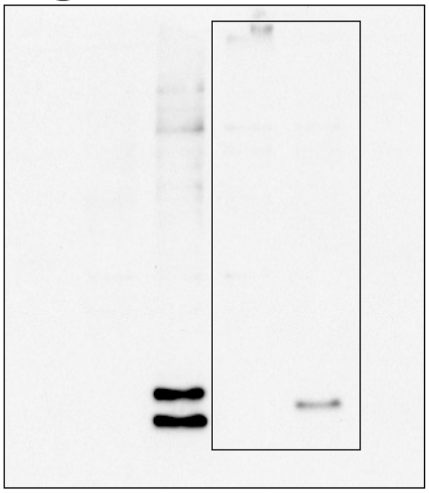

IB:DDK

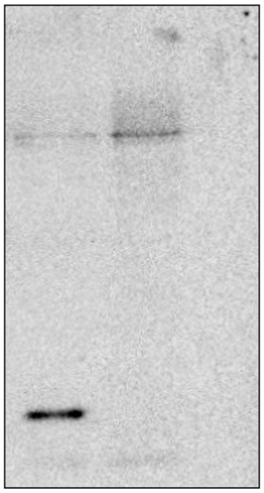

IB:COX20

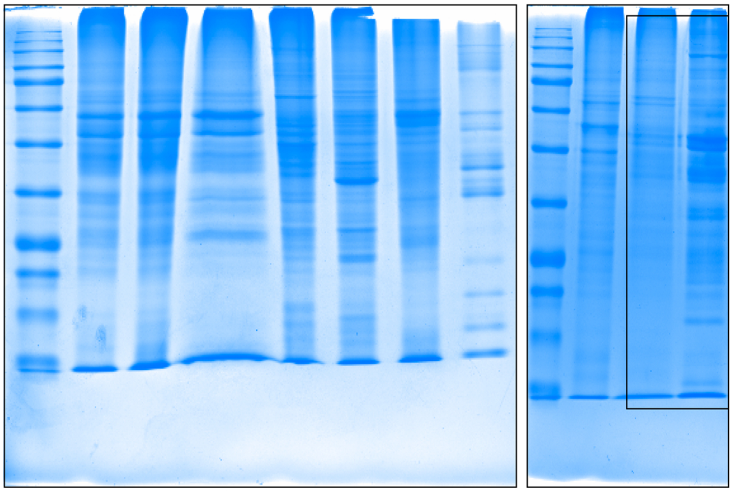

Coomassie Blue

Fig. 5d

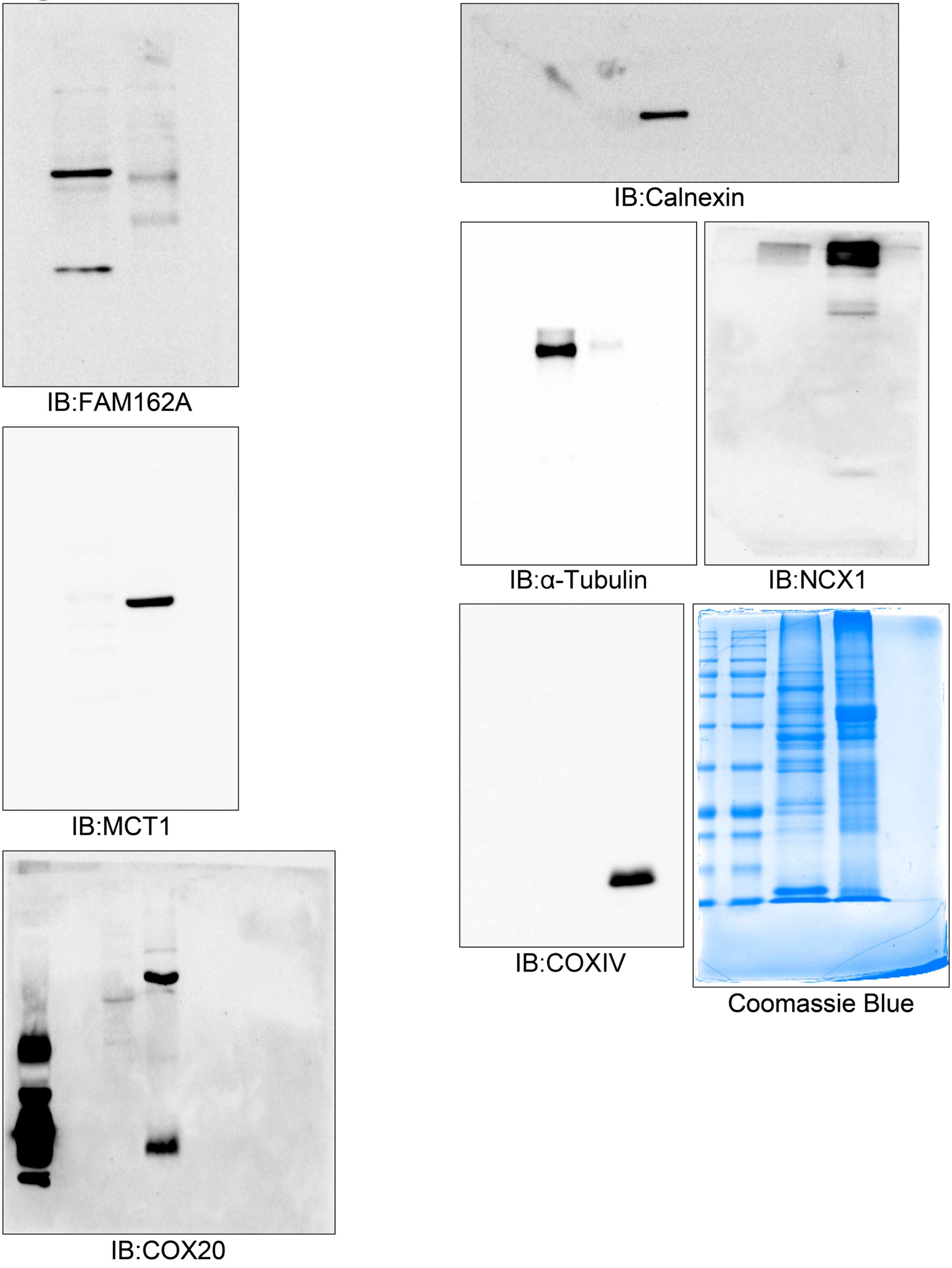
IB:FAM162A
IB:MCT1
IB:COX20
IB:Calnexin
IB:α-Tubulin
IB:NCX1
IB:COXIV
Coomassie Blue

Fig. 10a

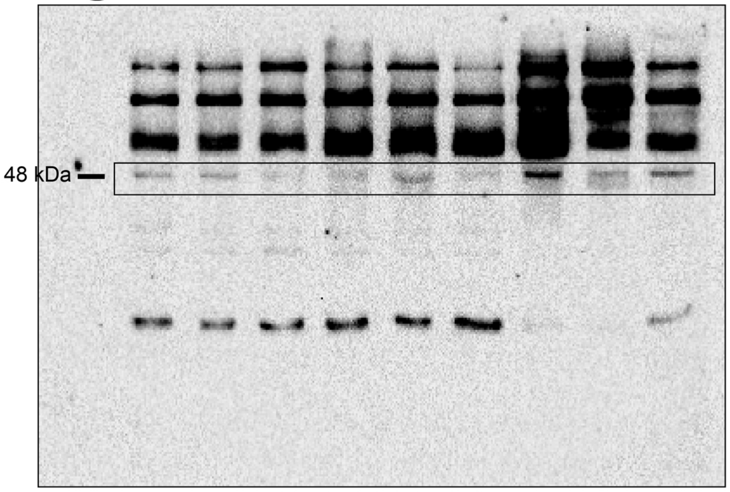

IB: FAM162A

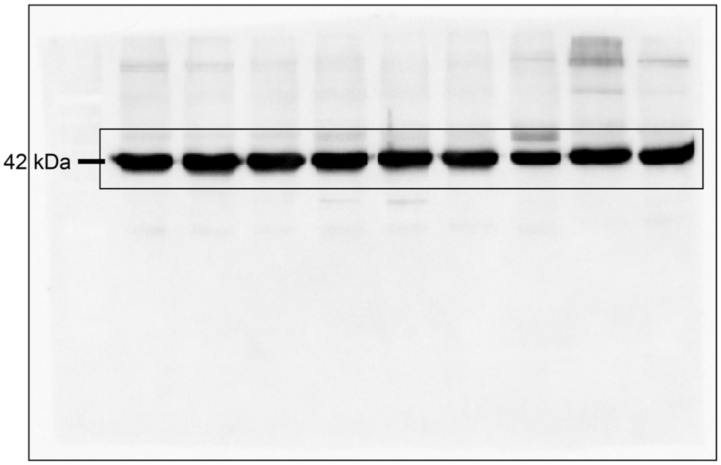

IB: α-actin

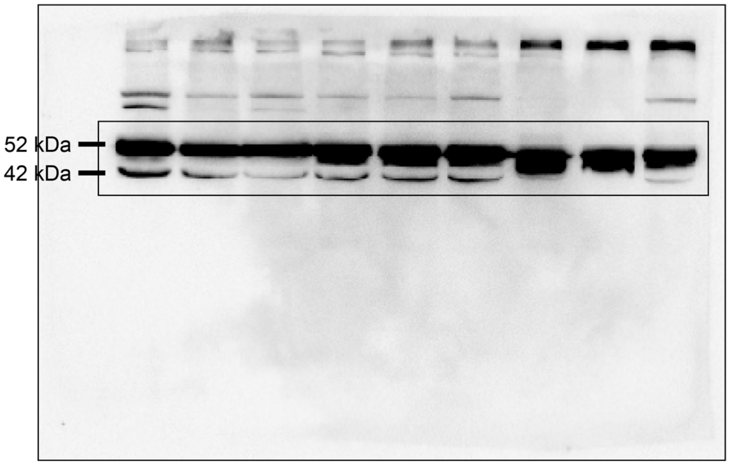

IB: MCT1

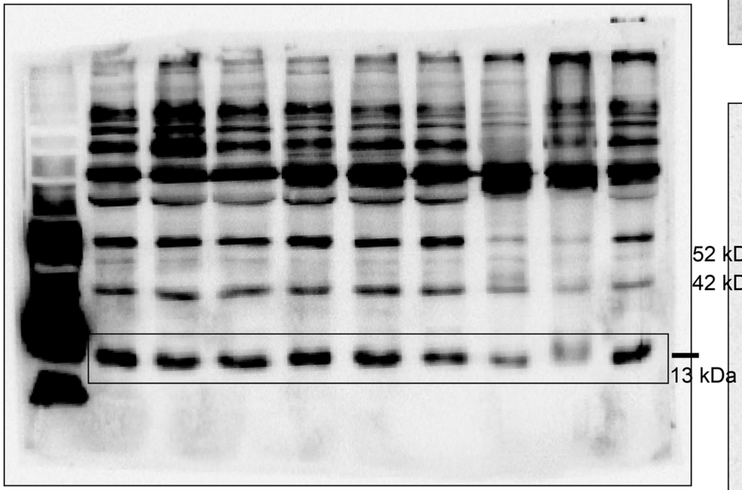

IB: COX20

Fig. 10b

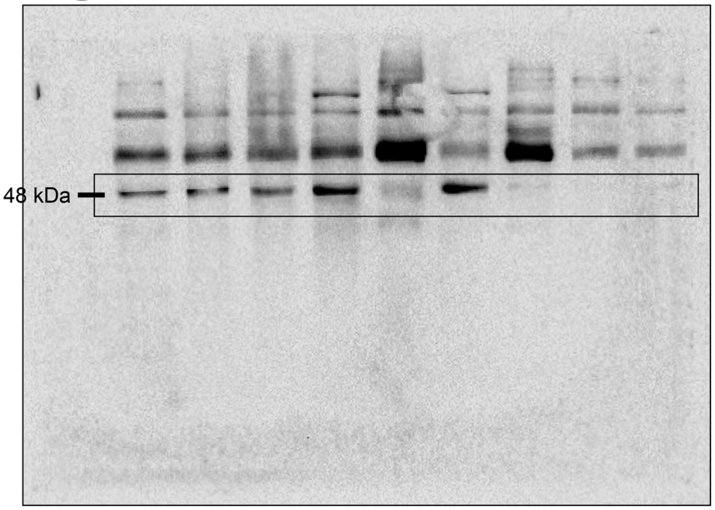

IB: FAM162A

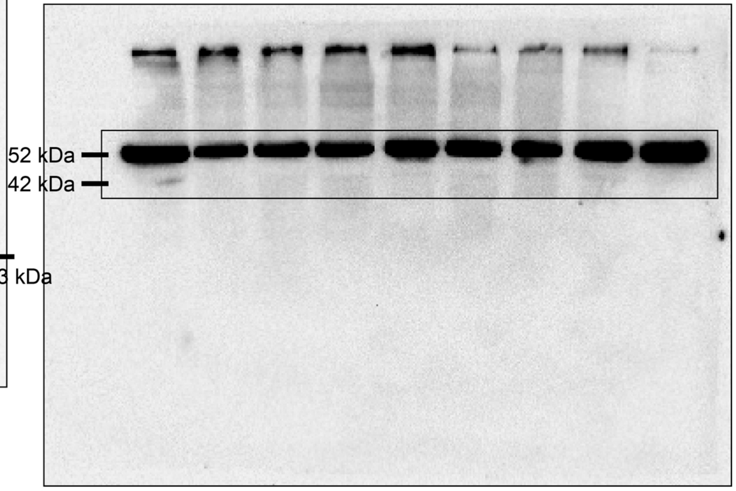

IB: MCT1

Fig. 10b cont.

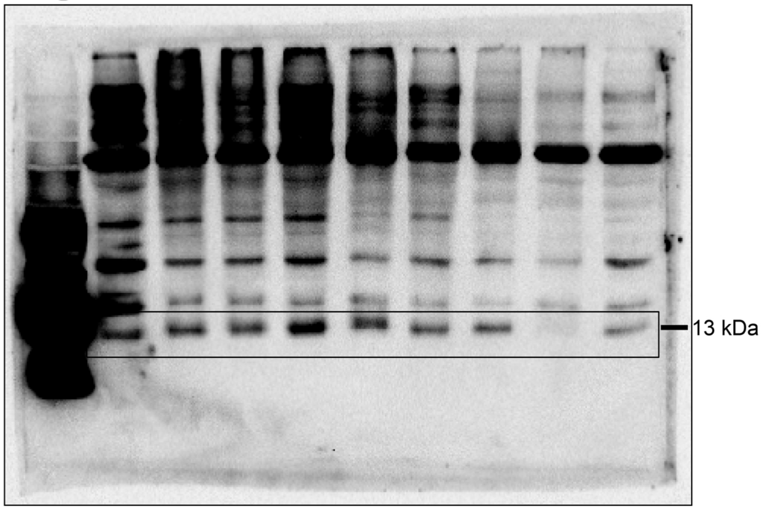

IB: COX20

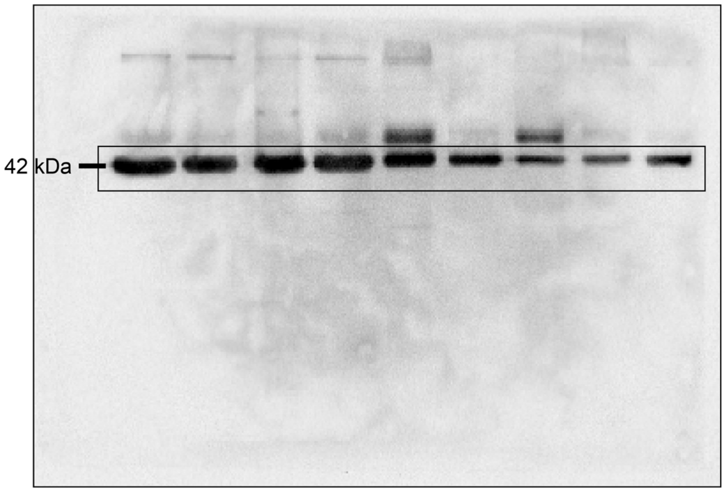

IB: α-actin

Supplemental Figure 9. Original uncropped immunoblots.
